# Why Homochirality in Biology? Coherent Water as a Morphogenetic Dynamic Mold Down to the Molecular Level: A QED Perspective

**DOI:** 10.3390/e28101065

**Published:** 2026-09-27

**Authors:** Paolo Renati

**Affiliations:** World Water Community, NL-3029 Rotterdam, The Netherlands; paolo.renati@gmail.com

**Keywords:** QED coherence, biological water, confined coherent modes, chirality, helicity, spin and orbital angular momentum, geometric phase, optical torque, morphogenesis

## Abstract

In previous works, it has been highlighted how the confinement of electromagnetic (EM) coherent modes—arising from their coupling with the water connectome of living matter—acts as a self-emergent ‘mold’ that spatially organizes molecules, membranes, vesicles and larger cellular structures through ponderomotive and dielectrophoretic forces: the standing waves of the self-trapped fields pattern matter into nodes and antinodes—as in cymatics—driving the template-free self-assembly of compartments from the nano- to the macroscale. In this perspective report, we extend that picture and propose specific physical mechanisms by which the same confined EM modes may act as the agent of one of the deepest levels of morphogenesis: the handedness/chirality of biomolecules. We first recall that the chiral content of light is not captured by polarization (spin angular momentum, SAM) alone: the true measure of the chirality of light is the helicity (ℋ), whereas the orbital angular momentum (OAM), carried by helical-wavefront (Laguerre–Gaussian-like) modes, governs global rotational transformations. We then show that (i) the geometric (Berry) phase implied by the chiral light–matter coupling is the quantum manifestation of optical rotation, both being governed by the same molecular pseudoscalar—the Rosenfeld rotational strength—that builds the mixed electric–magnetic polarizability responsible for chiral optical forces; (ii) the decohered one-dimensional (1D) lanes generated by the Anderson–Higgs–Kibble mechanism for the self-focusing of the fields traveling through the coherent aqueous medium are themselves optical vortices carrying a quantized flux, and, therefore, natural cavities for OAM-carrying modes whose topological charge selects, through an optical OAM torque, the handedness of supramolecular helices (like α-helices, as well as more complex geometries such as several collagen types); (iii) the helicity gradient developed at the boundaries of coherence domains acts as a ‘chiral mold’ that breaks the energy degeneracy of the ‘enantiomer + field mode’ pair and exerts an enantioselective SAM torque on point-like species (amino acids and sugars), lowering the activation barrier of the preferred enantiomer. Although small, this chiral bias operates within the coherent, low-entropy medium of biological water, being thus fueled and protected against thermal noise. We conclude that the homochirality of life is a direct and necessary consequence of the QED coherence of the aqueous matrix.

## 1. Introduction

Light, commonly perceived as a simple energy carrier or detecting probe, reveals a surprising structural and functional richness when analyzed with the tools of modern physics. In addition to its ability to transfer energy and momentum, EM radiation has intrinsic degrees of freedom related to its wave and quantum nature: spin, helicity and orbital angular momentum (OAM) [1], plus other quantities, known as Zilch tensors (see further details about this in Appendix A). These properties are not mere mathematical artifices but measurable physical quantities that govern the interaction between light and matter in a fine and powerful way, especially in biological contexts [2,3].

In recent decades, research has shown that these characteristics of light can be exploited not only to manipulate microscopic objects—such as for optical tweezers, chiral Stern–Gerlach deflector, or chiral diffraction grating [4,5]—but also to discriminate and separate chiral molecules, i.e., non-overlapping and mutually specular molecules, such as our hands (chiral). This ability is of fundamental importance for life, since biological systems are homochiral: they use only levorotatory amino acids and right-handed sugars. The crucial question then becomes: what is the physical mechanism that allows an EM field to “feel” and interact with the chirality of a molecule? And, even more profoundly, how can a complex system such as a living cell exploit these properties to guide biochemical synthesis to a specific enantiomer?

This section aims to answer these questions, integrating the knowledge from three deeply interconnected lines of research: (1) the physics of the angular momentum of light, (2) the quantum field theory applied to condensed matter, and (3) the QED vision of biological water as a coherent and dynamic matrix that organizes every molecular encounter—as well as the consequent biochemical reactions and assembly of products—in a non-random way. By merging these theoretical threads, it is possible to get an—at least semi-quantitative—idea that explains how the EM fields associated with the coherent oscillations, yielding the biological self [6], could act as real (fed-back) agents of biochemical syntax [6,7].

Furthermore, QED coherence in biological water provides a consistent physical basis for understanding how entropy in living systems is also minimized for the degrees of freedom related to chirality. In this regard, it is shown how coherence domains and coupled confined EM modes that act as multiscale ‘Maxwell’s demons’ that minimize local entropy and maximize the energy gap, non-equilibrium thermodynamics, information processing, and emergent phenomena are enriched [7].

## 2. The Angular Momentum of Light: Beyond Polarization

Traditionally, the interaction between light and matter has been described in terms of photon number (i.e., radiation intensity) and energy. However, light possesses momenta, namely, two distinct types of basic angular momentum, each with different physical implications [1,8].

### 2.1. Spin Angular Momentum (SAM) and Polarization

Angular spin momentum (SAM) is associated with the circular polarization of light. A photon with a right circular polarization (RCP) carries an SAM of (+ℏ), while one with a left circular polarization (LCP) carries a (−ℏ). This angular momentum is an intrinsic property of the photon, related to the local rotation of its electric-field vector around the direction of propagation [1,6].

However, although circularly polarized light seems to be the ideal candidate for interacting with chiral molecules, SAM is not a good measure of the chirality (*handedness*) of the light itself. In fact, under a parity reversal operation (such as a mirror reflection), the chirality of an electromagnetic wave changes sign (a right helix becomes left), but its spin (SAM) does NOT change. This is because the spin describes the intrinsic rotation of the electric field, not the geometry (possibly helical of the wavefront, which is global). Consequently, SAM cannot be the only quantity responsible for the interaction with molecular chirality, nor the most relevant.

It is important to ask a subtle question: how can a multitude of chiral molecules randomly oriented in a liquid solution, for example, produce the same angle of rotation to the plane of polarization of all photons with which they interact? This phenomenon, clearly, concerns the SAM of photons, which—as mentioned above—is associated with the polarization state, which does not change under parity inversion, because it is related to the intrinsic rotation of the electric field.

The reason why a solution can rotate the polarization of all photons uniformly lies in the difference between local and global transformations (see an intuitive sketch Figure 1):

- Local Property (Angular Spin Momentum, SAM): The optical activity of a molecular species is a *local* property. It derives from the non-superimposable structure of helical molecules or enantiomers and is a local effect as the rotation of the electric-field vector can be applied to any point within the beam regardless of its spatial position. This allows the medium (the solution) to act uniformly over the entire wavefront of the light that passes through it.
- Global Property (Orbital Angular Momentum, OAM): Conversely, a rotation associated with orbital angular momentum is a *global* transformation; it requires the rotation of the entire image structure around a specific axis of the light beam. The molecules in solution, acting locally, cannot coordinate a macroscopic rotation of the entire beam (which would require knowledge of its central axis), but they can act on the polarization (spin) of each photon individually wherever it is located [8].

### 2.2. Helicity: The True Measure of the Chirality of Light

Unlike the optical spin angular momentum, which does not change sign under a complete parity reversal (since it reverses both the orientation and the direction of propagation), there is another, much more important (although less known) physical quantity (a momentum as well) which is the helicity (H). It represents the projection of the spin of the photon along its direction of motion, expressed by the wave vector and is defined as

(1) $$H=S\cdot \hat{k}$$

where S is the spin, and $\hat{k}$ is the wave unit vector.

For a monochromatic plane wave, the helicity numerically coincides with the SAM, but conceptually it is different, because helicity is related to a fundamental symmetry of Maxwell’s equations and is a Lorentz-invariant quantity [10,11]. However, any conserved angular momentum (such as helicity) is associated with rotational symmetry, according to Noether’s theorem [9]. The conservation of helicity derives in fact from a specific “intrinsic” rotational symmetry that does not concern the spatial rotation of the entire beam (relative to the orbital angular momentum) but concerns a transformation that rotates the vectors of the electric (***E***) and magnetic (***B***) fields into each other (and vice versa) around the direction of the wave vector ($\hat{k}$). In mathematical terms, (in units, with c=1), this transformation is expressed as

(2) $$\left\{\begin{array}{c} E\to E+\theta B\\ \;B\to B-\theta E \end{array}\right.$$

where *θ* is a pseudoscalar adimensional angle (physically, *θ* does not describe a geometric rotation in three-dimensional space but rather parametrizes a ‘phase rotation’ in the abstract internal space of the field that mixes the vectors ***E*** and ***B*** into one another, whilst preserving the total energy; *θ* is defined as a pseudoscalar because it reverses its sign under spatial inversion (parity operation), thereby inextricably linking this symmetry to the intrinsic chirality of light).

This symmetry embodies the concept of ‘electromagnetic democracy’, placing electric and magnetic fields on an equal footing: the EM energy $E^{2}+B^{2}$ and the helicity remain the same ones. The conservation of helicity serves as a fundamental link between rotational symmetry and optical chirality, since helicity is, simultaneously, an angular momentum resulting from a symmetry of Maxwell’s equations and a rigorous measure of the ‘true’ chirality (handedness) of light [8,9]. ‘True chirality’—as introduced by Laurence Barron, in 1986 [12,13]—means that the enantiomeric states are interconverted by spatial inversion (parity, p) and not by temporal inversion (time, t), that is, when there is a pseudoscalar p-odd/t-even. See Section 5 and Appendix A for details.

To be more specific, the connection between rotational symmetry and optical chirality occurs through two key points:

- While the conservation of spin is associated with the rotation of field vectors in physical space (spatial rotation), the conservation of helicity is associated with the ‘dual’ rotation of vectors ***E*** and ***B*** around the direction of propagation;
- It is precisely this conserved quantity (helicity), resulting from the rotational symmetry of the fields, that determines the chiral light–matter interactions (see in the following). For example, in natural optical activity or chiral optical forces, the interaction energy depends explicitly on the helicity density of the field (i.e., how many revolutions its vector makes, around the direction of propagation, in a given direction (+ or −) per unit of length traveled).

In summary, the conservation of helicity implies that the chirality of light is not an elementary concept but deeply rooted in the fundamental rotational symmetries of electromagnetism. However, it is clearly distinguished from the spatial symmetries that govern spin and orbital angular momentum [9].

The interaction energy between an electromagnetic field and a chiral molecule depends on the product of the molecular chirality and the helicity density of the EM field. This explains standard phenomena such as optical rotation: a right-handed molecule interacts differently with light with positive helicity than with one with negative helicity, causing a difference in the refractive index (circular birefringence) for the two circular components (with which linear polarization can be represented more consistently), and, consequently, there is a phase shift between these circular components of the polarization at each point of the beam and a consequent change in rotation of the polarization plane.

### 2.3. Orbital Angular Momentum (OAM) and Global Beam Structure

The third item is given by the other basic momentum: a quantity, now global, called orbital angular momentum (OAM), associated not with polarization but with the spatial structure of the beam. The operator of the orbital angular momentum along the axis of propagation is defined as [8]

(3) $$L_{z}=-i\hbar (\frac{\partial }{\partial \varphi })$$

where φ is the azimuthal angle in the cylindrical coordinates system $\left(r,\;\varphi ,\;and\;z\right)$, it is the ‘angular position’ around the z-axis (its dimensions are [rad]). This operator acts on the spatial structure of the beam. When applied to a mode whose azimuthal dependence is $e^{il\varphi }$ (where φ is the azimuthal coordinate and l is an integer defined here below), it yields the eigenvalue equation, $L_{z}\;e^{il\varphi }=l\hbar \;e^{il\varphi }$, showing that lℏ is the measurable OAM per photon; *ℓ* is, therefore, the quantum number labeling the eigenmodes of $L_{z}$. The integer (l) is called *topological charge* because it is the *winding number* of the optical phase (Φ) (not to be confused with the azimuthal coordinate φ, since *Φ = Φ*(*r*,*φ*,*z*,*t*) and for a Laguerre–Gaussian mode $\Phi (r,\varphi ,z,t)=l\varphi +kz-\omega t+\Phi_{rad}(r)$) around the on-axis phase singularity (the optical vortex), where dl is a space length [m], and C is any closed transverse loop encircling the axis; l is defined by the line integral:

(4) $$l=\frac{1}{2\pi }\oint_{C}\nabla_{⊥}\Phi \cdot dl\;\;\in \;\;Z$$

Being a winding number, l cannot change under any continuous deformation of the beam: it is a topological invariant, which is precisely what makes it a robust ‘label’ for the mode [14,15]. $\nabla_{⊥}$ denotes the transverse gradient (the projection of ∇ onto the plane orthogonal to the propagation axis), i.e., the local transverse wavevector of the phase; its azimuthal component (ℓ/r) encodes the helical skew of the wavefront, while the longitudinal part (∂Φ/z = k) carries no topological content.

Laguerre–Gaussian (LG) beams [15,16] are a paradigmatic example; their wavefront has the shape of a helix, described by an azimuthal phase term $\left(e^{il\phi }\right)$ and each photon in such a beam carries an OAM having eigenvalues equal to lℏ. More precisely, the sign of ℓ fixes the handedness of the screw-like wavefront (right- vs. left-handed helix), whereas its modulus (|ℓ|) counts the number of 2π phase windings accumulated around the axis, i.e., the number of intertwined helical wavefronts [15]. The axial periodicity of this structure is set by the wavelength in the medium, λ: each individual helix advances by |ℓ|·λ per full 2π turn—equivalently, it rotates by 2π/|ℓ| per wavelength traveled—so that the whole |ℓ|-helix pattern reproduces itself after a translation of one wavelength along the propagation direction (as it will be shown in Section 4.3, this is what happen within the decohered lanes by the EM modes, and λ is the one of the traveling photons in there). This point will be reconsidered in Section 5.1 to deepen how this is linked to the conservation of the angular momentum ($J_{z}$), see Appendix B.

Thus, as we said, the SAM is manifested as polarization, and it is associated with the vector nature of the electric field and, in quantum theory, is related to circular polarization, where each photon carries a spin of ±ℏ. In contrast, the OAM is associated with the modal structure of the beam and the spatial shape of the wavefront. Beams carrying OAM possess helical (screw-shaped) wavefronts described by the azimuthal phase term seen above. Because of this phase dependence, the beam axis has a *phase vortex* (a central singularity where the intensity is zero) [16].

Light modes with OAM, such as LG beams, are generated and transformed by exploiting precise geometric relationships and analogies with polarization (SAM). Orbital angular momentum beam generation is commonly performed by converting standard laser beams that do not possess OAM.

The Hermite–Gaussian (HG) modes, typical of many lasers, for instance, do not possess orbital angular momentum but can be mathematically described as a superposition of pairs of “counter-handed” LG modes, having identical and opposite circular polarizations. It has been experimentally demonstrated that the use of cylindrical lenses (which focus the wave along a single direction) allows these beams to be generated by introducing a phase shift of π/2 between them and, thus, generating an LG mode, endowed with OAM, starting from an HG mode [8,9].

Just as a waveplate introduces a phase shift between orthogonal polarization states, a cylindrical lens mode converter acts the same way for OAM. The appropriate Jones matrix [9] for a cylindrical lens mode converter is identical to that of a waveplate; for example, a converter that acts as a “half-wave plate” inverts the image and is optically identical to a Dove prism (https://www.youtube.com/watch?v=-wFcgyIpnYI, accessed on 18 September 2026). The transformation equivalent to polarization rotation (optical activity) is less intuitive for OAM. While optical activity rotates polarization locally at each point, the equivalent transformation for OAM is a global rotation of the image (of the mode) around the beam axis.

OAM has distinct mechanical effects from SAM. While the SAM rotates a particle on itself, the OAM makes it orbit around the axis of the beam. This distinction is crucial. The transformations that act on SAM (such as the rotation of polarization through optical activity) are local: they occur independently at any point in space. On the contrary, again, the transformations that act on the OAM are global: they require a rotation of the entire structure of the beam around its axis. This fundamental difference explains why an optically active material, while rotating the polarization, has no effect on the OAM of a beam.

OAM also offers a technological advantage over digital-information-encoding capability: while spin has only two states (l=±1), OAM has a theoretically infinite number of states (l= 0, ±1, ±2, …). This makes it ideal for high-capacity optical communications, where each value of l can encode a distinct information channel [6] and, recently, it has been exploited as a basis for having manifold-states qbits differentiated from binary ones in a new prototype of quantum computing [17,18,19].

## 3. Light–Matter Entanglement and the Berry Phase

The interaction between light and chiral molecules is not a simple classic collision. At the quantum level, the photon and the molecule form an entangled system. The absorption of the angular momentum of light—for the law of its conservation—induces a mechanical rotation in the molecule, which, in turn, generates a geometric phase, known as Berry phase. This phase does not depend on the time or energy exchanged but on the geometry of the molecule’s rotation path [20].

In fact—to be a little more quantitative—when a quantum system undergoes a cyclic evolution in a parameter space (R) (here, the orientation angles of the molecule with respect to the field vectors or, equivalently, the polarization state on the Poincare sphere [1,9]), the total phase acquired by its state splits into two physically distinct contributions:

(5) $$\delta =\delta_{dyn}+\gamma_{B}$$

The *dynamical* phase, $\delta_{dyn}$, depends explicitly on the elapsed time (T) [s] and on the instantaneous energy eigenvalue ($E_{n}\left(t\right)$) [J]:

(6) $$\delta_{dyn}=-\frac{1}{\hbar }\int_{0}^{T}E_{n}\left(t\right)\;dt$$

The *geometric* (Berry) phase ($\gamma_{B}$) is given algebraically by the line integral of the Berry connection, A(R)=i⟨ψ(R)∣∇R ψ(R)⟩, along the closed path (C) [21,22]:

(7) $$\gamma_{B}=i\oint_{C}\left\langle \psi \left(R\right)|\nabla_{R}\psi \left(R\right)\right\rangle \cdot dR$$

where $\psi \left(R\right)$ is the instantaneous normalized eigenstate of the coupled photon–molecule system (dimensionless); R is the vector of adiabatically varied parameters defined by the orientation angles—polar, β and azimuthal (φ) (both in [rad] in the unitary Poincare sphere): in practice, these two angles define the orientation vector on a unitary sphere, which defines the relative orientation between the chiral axis of a molecule and the axis of the helicity (equal to that of propagation) of the field; $\nabla_{R}$ is the gradient in the parameter space (R) in [rad^−1^]. Since Equation (7) contains neither t nor $E_{n}$, $\gamma_{B}$ is invariant under any re-parametrization of the speed at which the path is traversed: it depends only on the geometry of the rotation path (C). For a cyclic rotation, Equation (7) reduces to the solid-angle rule:

(8) $$\gamma_{B}=-\;m_{j}\;\Omega_{C},\;with\;\Omega_{C}=\oint_{C}\left(1-\cos\beta \right)\;d\phi$$

where $m_{J}$ is the projection (in units of ℏ) of the total angular momentum of the participating state branch onto the rotation axis: $m_{J}=\sigma$ for a spin-redirection loop of the wavevector, $m_{J}=\sigma /2=\pm 1/2$ for a polarization loop on the Poincare sphere (with σ=H/ℏ), and $m_{J}\in \{-J,\ldots ,+J\}$ for a molecular rotor in the state $|J,m_{J}\rangle$ [20,21,22,23]. For a molecular rotor, $m_{J}$ is the relevant rotational quantum number. $\Omega_{C}$ is the solid angle subtended by the closed rotation path on the unit sphere: here, the spherical coordinates (β and ϕ, polar and azimuthal angle, respectively) do not refer to arbitrary geometric axes, but describe the relative orientation between the molecule’s intrinsic chirality vector (${\overset{⃑}{n}}_{chiral}$, defined by the cross product of its electric ($\overset{⃑}{\mu }$) and magnetic ($\overset{⃑}{m}$) transition dipoles ($\overset{⃑}{\mu }\times \overset{⃑}{m}$)), and the wavevector/helicity axis ($\hat{k}$) of the confined EM mode and m is the projection (in units of ℏ) of the angular momentum exchanged along the rotation axis [15,20,21,22,23,24,25]. Thus, the Berry phase is the real quantum manifestation of the optical rotation: the measurement of the entanglement (the “concurrence”) between photon and molecule is directly proportional to the cosine of the optical rotation angle.

### A Few Examples of Technologies Based on the Chirality of Light

Before linking these physical processes to biological syntax and the stringent enantiomeric selectivity of which living matter is capable, it is worth mentioning that these properties of light have been used sophisticatedly in the technological field, leading to the development of revolutionary methods for the manipulation of matter at the molecular level. If we make sure that the helicity of light is not uniform in space and that it has a gradient, then we generate not only a difference in energy but a real force. This chiral optical force acts in the opposite way on specular enantiomers. Imagining a helicity gradient, the right molecules are pushed in one direction, while the left molecules are pushed in the opposite. This principle has allowed for the conception of devices such as the chiral Stern–Gerlach deflector [4]. Similar to the original experiment that separated atoms based on their electron spin using a magnetic field gradient, this device uses a helicity gradient to separate molecules based on their chirality.

Another example is the discriminating chiral diffraction grating, a pattern of structured light with helicity gradients that, crossed by a racemic beam of molecules, is able to impart an optical force that deflects the two enantiomers at different angles, allowing for their spatial separation [5].

More relevant is the experimental proof of the ability of Laguerre–Gaussian light beams not only to trap or selectively drift chiral molecules but to make them spin/rotate. Until 1936 (Beth’s experiment [26]), it was known that circular polarization (SAM) could cause a microscopic foil to rotate. But the OAM (the twist of the wavefront) had remained purely theoretical. In 2001, Paterson and his colleagues [27] carried out a brilliant experiment: they created a Laguerre–Gauss beam (endowed with OAM, i.e., with a corkscrew-shaped wavefront) and used it to trap microparticles of copper oxide in an optical-tweezers-like setup. As the particles absorbed the light, they also absorbed the orbital angular momentum (ℓℏ) of the photons. The result is that the particles began to spin rapidly round and round under the microscope’s lens. The researchers called it the ‘optical spanner’ because they demonstrated that topologically complex wavefront light does not merely push or pull the particles (as in Ashkin’s classic optical tweezers) but applies genuine mechanical torque capable of ‘screwing’ matter, just as a spanner turns a bolt. This proved that the OAM could exert macroscopic mechanical torque. In Section 5.1, we will deepen this aspect.

## 4. QED Coherence in Living Matter and Role of the EM Fields in Morphogenesis and Topology: A Preliminary Summary

So far, we have discussed the light–matter interaction in a generic context. However, in biological systems matter is not a collection of molecules isolated in a passive solvent. As we have extensively illustrated in previous works [6,7,28,29,30,31], the main constituent of life is water, and its nature is not that of a simple fluid but the one of a coherent connectome. Let us revisit some key concepts here to link them to the theme of homochirality considering the analysis conducted so far. Living matter is not a random molecular soup but a highly structured and inhomogeneous medium in which coherent electromagnetic (EM) oscillations establish stable interference patterns and resonance-ruled biochemical encounters.

It is worth stressing that the QED/QFT framing is not required to yield the optical description of the light–matter coupling—helicity, SAM, OAM and zilch are well-defined properties of the electromagnetic field also outside a coherent medium—but it is needed by a truthful description of the condensed phase of biological (water-based coherent) medium itself. Only in a (super)coherent aqueous connectome do the confined EM modes acquire the integra, system-wide capacity to govern where, when and in which chiral-form hetero-molecular syntheses occur; synthesis, indeed, is restricted to the regions (CD boundary surfaces and decohered lanes) in which precisely those modes percolate/decay or propagate, and the associated field (and its properties) gradients (∇A^2^ and ∇h) act as selecting agents on the whole assembly process. At a certain stage, we could state that the physical causal relationship is somehow ‘circular’: pre-existing chiral structures template the chirality (helicity sign) of the water connectome and of its confined modes, which, in turn, condition the handedness of the molecular products synthesized within that context.

In this section, we summarize the main aspects which let us understand how coherence plays a ‘demiurgic’ role both morphologically and biochemically. Of course, homochirality is one of the most delicate and fundamental ambits where these two sides are intertwined with one another; we will focus on this latter point in Section 5.

### 4.1. The Necessity of a Quantum Field Theoretical Framework

Living matter challenges the descriptive capacity of classical physics and semi-classical quantum mechanics (QM) in fundamental ways. The extraordinary precision, efficiency, and timing of biochemical processes; the emergence of morphogenetic patterns from a single zygote; the establishment of homochirality; the existence of ordered reaction cycles (e.g., Krebs and Calvin); and the capacity for adaptation and teleological behavior cannot be accounted for within a framework that admits only a unique ground state and treats molecular encounters as stochastic, short-range events [3,29,30,31,32]. In the conceptual framework of QM, and in accordance with von Neumann’s fundamental theorem [33], a unique vacuum state exists for any ensemble of interacting particles, thereby precluding the description of phase transitions: the very processes through which living matter emerges and sustains itself [34,35,36,37,38].

Quantum field theory (QFT), by contrast, admits a multiplicity of unitarily inequivalent vacuum levels and provides the formal apparatus to describe spontaneous symmetry breaking (SSB), dissipation, and the dynamical emergence of order as a condensation of Nambu–Goldstone (NG) bosons into the ground state [39,40,41]. Within QFT, the field (including the matter field) holds ontological primacy: the particle, or “quantum”, is a derivative excitation of the field, and the phase of oscillation—rather than the number of quanta—is the paramount observable [34,35]. This conceptual shift, from the corpuscular (first quantization) to the field-theoretic (second quantization) description, is a ‘*conditio sine qua non*’ for understanding how long-range correlations, coherent dynamics, and ordered configurations arise in condensed and living matter [7,30,31,42].

In SSB, the symmetric properties of the microscopic dynamics imply the presence of fields which, distributed throughout the system, cause the formation of ordered configurations of the elementary components. The NG quanta associated with such dynamics are bosons that condense in the vacuum, mediating phase correlations over large distances and constituting the structural elements of the ordered state [7,9,14]. Crucially, these correlation quanta cannot be ‘detached’ from the system: phonons do not exist outside a crystal, just as the coherent modes of living matter do not survive the disruption of the organism. The NG quanta are, thus, identified with the function of the specific order for which they are responsible, dissolving the Cartesian dualism between structure and function [31,40].

### 4.2. QED Coherence

Keeping in mind that the relation between electric (***E***) and magnetic (***B***) fields and potentials (V, scalar; ***A***, vector) is

(9) B=∇×A

(10) $$E=-\frac{\partial A}{\partial t}-\nabla V$$

and that the relation between the scalar/vector potential and the phase of oscillation, in QED, is (MKS unit system):

(11) $$V=\left(-\frac{h}{2\pi e}\right)\frac{\partial \varphi }{\partial t}$$

(12) $$A=\left(\frac{h}{2\pi e}\right)\nabla \varphi$$

It is easy to define the dynamic processes underneath the dislocation of matter oscillators within a fully (super)coherent medium (‘super’ because it is a medium in which coherence is in force on several degrees of freedom, and a nested hierarchy of coherences implies a full dependence of the ones on the others [7,31,43,44]).

The principles of SSB, applied to liquid water, provide a fully consistent picture of both plain water and living matter. Once a critical threshold density is reached (0.32 g/cm^3^ below 373.15 K at 1 atm), SSB induces the condensation of water molecules into coherently coupled domains [29,45,46,47]. According to QED [48,49,50], part of the molecules in liquid water are organized in collective, coherent vibrational domains—termed coherence domains (CDs)—whose size corresponds approximately to the wavelength of the coupled electromagnetic mode [46,47,48,49,50]. For the electronic oscillation between the sp^3^ hybrid orbital and the 5d excited level (ΔE = 12.07 eV), the ideal CD radius is approximately 100 nm, containing on the order of 10^6^–10^7^ molecules [29].

The coherent ground state is energetically lower than that of incoherent, isolated molecules. The energy gap (approximately 0.16–0.20 eV for the sp^3^–5d oscillation at physiological temperatures) expresses the thermodynamic stability of the coherent state against decohesion agents, like thermal agitation [46]. Each molecule participating in the coherent dynamics possesses an electron that spends approximately 10% of its time at the 5d level, which lies at most 0.53 eV below the ionization threshold (i-th = 12.60 eV). This yields approximately 0.10 quasi-free electrons per coherent molecule, amounting to ~10^6^ quasi-free electrons per CD [29,47].

This EM field (namely, the quadri-vector potential (***A***)) is trapped within the CD region not only because, once the critical density has been reached, its oscillation time is renormalized by the same coupling with molecules; the time of oscillation of the free photon should be actually supplemented by the time spent within the molecules in the form of excitation energy. Consequently, the frequency of the EM field in the CD ($\nu_{CD}$) becomes smaller than the frequency ($\nu_{0}$) of the free EM field and the squared mass of the photon (m^2^) becomes non-zero (and negative), according to the Einstein equation and the well-known Anderson–Higgs–Kibble (AHK) mechanism or Kibble–Zurek dynamics, which will be described in [51,52,53].

The interesting aspect at this point of the manuscript is to note that—thanks to the AKH mechanism—the CDs act as self-generated total-reflectance cavities for the EM field: no external pump or cavity is provided from outside, as the self-trapped EM field is an intrinsic phenomenon arising from the coupling of the matter field with the radiative field, implying total internal reflection [29,50,54]. The coherent fraction of liquid water at room temperature and pressure has been calculated to be around 40% [29,45,46], but in biological matter—where hydrophilic surfaces, membranes, and ionic species further stabilize the coherent fraction—the whole water matrix is involved in several hierarchies of coherence (i.e., it is supercoherent [3,7]), which are modulated depending on the coupling with the environment and, thus, the typical condition of the water in a cell is a sol–gel state that is, in a meaningful sense, ‘solid’ [55,56].

### 4.3. Field Gradients at Coherent Boundaries and Self-Focusing: A Basis for 1D, 2D, and 3D Topologies in Living Matter

The emergence of ordered structures in living matter cannot be understood without first envisioning (i) the mechanism by which the EM potential becomes confined within the coherent aqueous matrix (for their massless component) and how electric and magnetic fields are segregated within the incoherent zones (as narrower as possible) to minimize the free energy of the system [34,54], on the one hand, and (ii) how the oscillation modes drive the biochemical pathways, on the other.

#### 4.3.1. AHK Mechanism and Genesis of One-Dimensional Structures

It is useful to specify better that in the genesis of CDs, as the numerical density of molecular oscillators per unit volume increases—as occurs during cooling or under pressure—the probability that a photon, previously absorbed and subsequently re-emitted by one molecule, excites a neighboring molecule approaches unity (100%). In this regime, the EM energy (the photon) spends part of its time as excited matter and part of its time as a free field. This alternation produces a temporal stretching of the oscillation and a consequent reduction (renormalization) in the field frequency. This mechanism is formally known as the AHK mechanism [51,52,53,54]:

(13) $$\nu \;⟶\;\nu_{\mathrm{CD}}=\nu -\frac{\partial \varphi }{\partial t}\;<\;\nu =\frac{c}{\lambda }$$

where φ is the phase of the coherent matter–field oscillation. The renormalized frequency ($\nu_{\mathrm{CD}}$) is strictly lower than the free-field frequency (ν= c/λ). Recalling the relativistic energy–momentum relation, E = hν, $E=mc^{2}$, and p = h/λ, one obtains:

(14) $$m^{2}c^{4}=E^{2}-p^{2}c^{2}=h^{2}\nu^{2}-\frac{h^{2}c^{2}}{\lambda^{2}}=h^{2}\left(\nu_{\mathrm{CD}}^{2}-\frac{c^{2}}{\lambda^{2}}\right)<0$$

Since $\nu_{\mathrm{CD}}<c/\lambda$ and $h^{2}>0$, necessarily, then, the quantity in parentheses is strictly negative, resulting in

(15) $$m^{2}<0\;\to \;mass:\;imaginary$$

This condition defines quasi-particles (field–matter coupled excitations) that cannot propagate freely: the photon is self-trapped within the molecular ensemble whose spatial extent is comparable to the photon’s own wavelength. This confined space–time region is right the abovementioned CD, within which all molecules oscillate in phase with one another and with the self-trapped EM field [29,46].

In the language of QFT, the dynamics of the system are described by a set of field equations hypothesized to contain all relevant observables. The fields represent the elementary components of the system—the electromagnetic field, the atomic and molecular constituents, their electric charges, and their dipole moments, all expressed as fields in the second quantization [34,35,36]. The dynamics is invariant under symmetry transformations (rotations, translations, and gauge transformations). However, under the action of an external or endogenous perturbation, the ground state (vacuum) of the system may cease to be symmetric. The symmetry is then said to be spontaneously broken: the system is driven into a non-symmetric state by its own internal dynamics, the perturbation acting merely as a trigger. The Goldstone theorem guarantees that long-range correlations are dynamically generated when symmetry breaking occurs. These correlations produce the ordering of the elementary components through collective modes (waves) that propagate without destructive interference, i.e., coherently. The quanta associated with these long-range correlation waves are the Nambu–Goldstone (NG) bosons, and the ordering is dynamically generated by their coherent condensation into the vacuum state [37,42]. The macroscopic order is described by the order parameter, a classical field (i.e., insensitive to quantum fluctuations) representing the coherent correlation field [35,54].

In a generic ensemble of water molecules, no preferential direction exists along which the molecular electric dipoles point or oscillate in phase: the system possesses full rotational symmetry. However, when a perturbation (an external or endogenous electric field, a variation in charge density or ionic currents, a shift in electric potential, or the presence of a surface bearing oscillating electric charges) triggers the alignment of molecular dipoles along a preferential direction, or induces their oscillations to become in-phase, the system reaches a state characterized by a polarization density ($P\left(r,t\right)$). The original spherical (rotational) symmetry is spontaneously broken. Polarization density plays the role of the order parameter, and the NG quanta are now the *dipole wave quanta* (DWQ) [57,58].

The crucial consequence of DWQ condensation is that the EM field propagating in regions where DWQ are coherently condensed acquires an *effective mass* (*M*) proportional to the polarization density ($P\left(r,t\right)$). This implies that the field (and its associated photons) no longer propagates as spherical waves but remains *focused* into ‘channels/lanes’ whose transverse dimension (*d*) is inversely proportional to *M*. In the spontaneous symmetry-broken state (SSB), a self-focusing propagation of photons, thus, emerges as a dynamical result of the coherent ordering of the medium [24,59].

If $A\left(x\right)$ is the EM potential penetrating the medium along the transverse direction x (while the propagation one is the *z* direction), the static damped solution describing its decay within the coherent medium is

(16) $$A\left(x\right)=A\left(0\right)\;e^{-cMx/\hbar }$$

where c is the speed of light in vacuum. The effective mass (M) is given by

(17) $$M^{2}=\frac{q_{m}^{2}\;P}{\delta }$$

where δ is the mean length of the dipole induced by the passing field, and P is the macroscopic average polarization of the medium defined as

(18) $$P=\frac{\varepsilon_{0}}{2\pi }\;\frac{\delta_{n}}{n_{0}}\;E$$

where $n_{0}$ is the refractive index of the medium (approximated as isotropic), $\delta_{n}$ is the perturbation effect on $n_{0}$ due to the propagation of the electric field (***E***) itself (a quasi-monochromatic wave propagating through an initially coherent medium), and $\varepsilon_{0}$ is the vacuum permittivity. Knowing that E = −∂A/∂t, the transverse cross-section (*d*, corresponding to the diameter (∅)) of the propagation channel is

(19) $$d\equiv \emptyset =\frac{\hbar }{c}\;\frac{1}{M}$$

This relation establishes that the narrower the channel, the larger the effective mass (*M*) of the field within the coherent medium. See an intuitive sketch of the 1D AHK self-focusing of the field and creation of molecular chains along the decohered lanes in Figure 2.

The behavior of EM fields within coherent biological matter is directly analogous to that of magnetic fields in Type-II superconductors. The expulsion of the field from the coherent system is known as the Meissner effect: fields are permitted to enter the coherent system only on the condition that they self-focus into flux lines constituting incoherent ‘cores’ or ‘lanes’ [57,58]. These decohered corridors, traversed by oscillating dipole currents, become the privileged pathways along which the field propagates and along which matter is subsequently organized.

In analogy with superconductors, the decohered zones by the passage of the field are internally traversed by currents arising from the oscillation of electric dipoles. The polarization current density is

(20) $$J_{P}\left(x\right)=\rho \left(x\right)\;u$$

where $\rho \left(x\right)$ is the charge density, and u=∂δ/∂t is the time variation (oscillation) of the dipole length (δ). Writing the wave function of the charge density as

(21) $$\sigma \left(x\right)=\rho \left(x\right)\;e^{i\theta \left(x\right)}$$

where $\theta \left(x\right)$ is the phase of the oscillation, and $\rho \left(x\right)$ and $\theta \left(x\right)$ are real functions, the polarization current density becomes

(22) $$J_{P}\left(x\right)=\frac{1}{M}\;\rho \left(x\right)\left[\nabla \theta \left(x\right)-q\;A\left(x\right)\right]$$

As a boundary condition, the current within the ordered (coherent) region must vanish, $J_{P}=0$, exactly as in superconductors. This yields the fundamental relation

(23) ℏ ∇θ=q A

Integrating (taking the circulation of) both sides of Equation (23) around the cross-section of the region along which the field propagates (i.e., an initially coherent region), as well as noting that the phase variation of the dipole oscillation must be an integer multiple of 2π (i.e., 2πn, with n an integer) because the dipoles are coherent, one discovers that the magnetic flux associated with the EM potential (A) is quantized (which corresponds to the Josephson effect [58,60]):

(24) ℏ∮∇θ⋅ds=ℏ 2πn=nh≡q∮bA⋅ds=q Φ

Therefore

(25) $$\Phi =n\;\frac{h}{q}$$

Each variation in the EM field corresponds to a discrete variation in the magnetic flux (Φ), confined along the decohered lanes (on one or a few degrees of freedom) traversed by the coherent dipole oscillations. The scalar and vector potentials are related to the phase, as defined before in Equations (11) and (12), and the EM potential (A) undergoes, for the maintenance of gauge invariance, a phase variation (coupling with the coherent DWQ):

(26) $$A\left(x\right)\;⟶\;A^{\prime }\left(x\right)=A\left(x\right)-\frac{1}{q}\;\nabla \varphi$$

Inserting the physical parameters of the coherent aqueous medium (at physiological conditions of room temperature and pressure) into Equation (19)—and given that more than 90% of the molecules in the cytosol are water [30], one can estimate that a field capable of decohering the medium must have an energy scale comparable to the electromagnetic mode that keeps the molecules coherent: E ≈13 eV [57,58,60]—one obtains [58]

(27) $$Mc^{2}\approx 13\;\mathrm{eV}\to \emptyset \approx 150\;Å$$

The value ∅≈150 Å is quite precisely comparable to the transversal size of cytoskeletal microtubules (outer diameter of ~250 Å; inner diameter of ~150 Å) [58].

The structures of the cytoskeleton—filamentous structures produced and maintained in the cytoplasm through metabolism—are, therefore, the dynamical consequence of the self-focusing of electric polarization waves in the cellular medium which behaves in a strongly nonlinear fashion. Indeed, in simple (non-coherent) liquids, the diameters of self-focused beams are of the order of microns, whereas the characteristic diameters of cytoskeletal filaments are a few hundred Å, approximately 30 times smaller. In the biological context, self-focusing is far stronger than in ordinary (non-coherent) materials precisely because all molecules are correlated over long range [57].

The AHK self-focusing mechanism, combined with the Meissner-like expulsion of fields from coherent domains and the quantization of flux into incoherent lanes, provides the physical basis for the generation of all one-dimensional (1D) structures in living matter. These include the following:

- Cytoskeletal filaments: microtubules (~250 Å outer diameter), actin filaments (~70 Å), and intermediate filaments (~100 Å);
- Molecular chains: polypeptide backbones, nucleic acid strands, and polysaccharide chains;
- Collagen fibrils and bundles: triple-helical tropocollagen molecules assembling into fibrils of ~500–3000 Å;
- Proteoglycan core proteins and glycosaminoglycan chains;
- Elastin fibers and fibronectin filaments in the extracellular matrix.

In each case, the self-focused EM field creates a decohered lane: a 1D incoherent (on that degree of freedom) corridor within the otherwise coherent aqueous medium. The intense field gradients at the boundary between the coherent domain and the incoherent lane generate strong ponderomotive and dielectrophoretic forces (as described in the following section) that attract, orient, and polymerize molecular species along the axis of the channel (for 1D topologies) or at the open and closed boundary surfaces (for 2D and 3D cases).

#### 4.3.2. Two- and Three-Dimensional and Ordered Biochemical Pathways

The self-trapping of the electromagnetic potential (***A***) within CDs generates strong spatial gradients at the interface between the coherent and incoherent fractions. These gradients give rise to ponderomotive and dielectrophoretic forces that act upon any charged or polarizable species present in the vicinity of the CD boundary. According to the laws of electrodynamics, these forces decompose into a static (frequency-independent) contribution and a dynamic (frequency-dependent) contribution [3,31,61].

The static ponderomotive force ($F_{pm}$, always repulsive, independent of frequency and the electrical charge sign) is expressible as

(28) $$F_{pm}=-\frac{Q^{2}}{M}\;\nabla A^{2}$$

where Q is the electric charge of the species under consideration, M is the mass of the electrically charged species, and ∇A^2^ is the spatial gradient of the square of the self-trapped electromagnetic potential (field) (***A***) within the CD. The minus sign indicates that the force is always repulsive (pushes the charges out of the CD). The intensity is proportional to the charge/mass ratio (q^2^/m): electrons, being ~2000 times lighter than protons, move significantly outward more than the latters, strongly polarizing the approaching molecules. This force is always repulsive, acts on any electric charge regardless of sign, and its intensity is proportional to the charge-to-mass ratio (q^2^/m). Since electrons are approximately 2000 times lighter than protons, they are displaced outward far more strongly than nuclei, resulting in a pronounced polarization of molecules approaching the CD periphery. This polarization renders molecules highly reactive, lowering activation thresholds and accelerating reaction kinetics [3,6,31].

The dynamic (frequency-dependent) component of the electrodynamic interaction force between a CD and an i-th species can be expressed as

(29) $$F_{\text{i-CD}}=C\;\frac{\omega_{\mathrm{CD}}^{2}-\omega_{i}^{2}}{{\left(\omega_{\mathrm{CD}}^{2}-\omega_{i}^{2}\right)}^{2}+\Gamma^{2}}\;\;\nabla A^{2}$$

where C is a constant, $\omega_{CD}$ is the instantaneous frequency of oscillation of the CD, $\omega_{i}$ is the natural (proper) frequency of the i-th species, and Γ is the damping coefficient. When $\omega_{i}$ is far from $\omega_{CD}$, the force is negligible, but when $\omega_{i}\to \omega_{CD}$ (resonance), the denominator is minimized and the force becomes strongly attractive (ideally divergent, in the limit Γ → 0), drawing the species strongly towards (+) or away from (-) the CD interface in dependence of its algebraic sign. This frequency-dependent force describes how a given atomic or molecular species interacts with the CD as a function of the match (or mismatch) between its proper vibrational frequencies [31,42,62].

The expression pertaining to the mediation role of the EM potential of water CDs on the chemical encounter between two molecular species (1 and 2) is written as

(30) $$F_{\mathrm{int}\;\text{1-}2}=C\;\frac{\omega_{1}^{2}-\omega_{2}^{2}}{\left[{\left(\omega_{1}^{2}-\omega_{\mathrm{CD}}^{2}\right)}^{2}-{\left(\omega_{2}^{2}-\omega_{\mathrm{CD}}^{2}\right)}^{2}\right]+\Gamma^{2}}\;\;\nabla A^{2}$$

where $\omega_{1}$ and $\omega_{2}$ are the natural frequencies of the two chemical species.

This equation governs the selective attraction or repulsion between two species in the presence of the coherent background field: only molecules whose frequencies are commensurable with the oscillation of the CD can meet at the interface, acting as a frequency ‘filter’ that orchestrates molecular encounters in ordered, non-random sequences (see Figure 3 and Figure 4).

This view reveals the profound reasons why ordered biochemical pathways are performed in biology, wherein at each step the right molecular (or ionic) species become selected partners. When the resonance-selected’ partners are attracted at CDs’ boundaries, they experience catalytic role of coherent water in the (mainly) redox reactions they are involved. The energetically ‘cheap’ quasi-free electrons of coherent water ceded to the reagents, as well as some EM quanta of energy (photons) and new products, at the right time and place are synthetized. This interplay between CD and molecular species implies that, at each reaction step, the oscillation frequency of the engaged CDs is shifted to new values, now able to get into resonance with new species (see Figure 4 for an intuitive idea) providing a consistent physical basis for the precise and efficient biochemistry in living matter (irreproducible in vitro).

The field gradients described above do not merely govern molecular encounters, they act as morphogenetic agents that shape the very topology and geometry of living matter. The study of how oscillating fields pattern matter in space is known as cymatics [64], and it occurs for both mechanical/acoustic and electromagnetic waves [65,66]. With EM waves, the mechanism is the same and is known as dielectrophoresis (DEP), which concerns the force developed by time-varying fields on polarizable particles depending on the frequencies, amplitudes, wave profiles, and dielectric properties of the particle and medium [28,67]. In other words, it describes the force exerted on a dielectric particle in the presence of a non-uniform electric field. The particle may also be neutral. The magnitude of the force depends on the dielectric properties of the particle itself and those of the medium in which it is immersed, as well as on the particle’s shape and size (in relation to the wavelength or frequency of the oscillating or non-uniform field). The Clausius–Mossotti relation rules these kinds of processes, relating microscopic dielectric properties (polarizability) to macroscopic electromechanical quantities (density and polarization) in the medium, where oscillators (also neutral) are dispersed and the EM waves propagate [67,68] (see Figure 5 for an example).

(31) $$\frac{\varepsilon -\varepsilon_{0}}{\varepsilon +2\varepsilon_{0}}=\frac{4\pi }{3}\;\frac{N_{A}\;\alpha \;\rho_{m}}{M}$$

where ε is the dielectric permittivity of the medium, ε_0_ is the vacuum permittivity, $N_{A}$ is Avogadro’s number, α is the average polarizability, $\rho_{m}$ is the mass density, and M is the average molar mass of the oscillating components.

The shape and the possible multi-layering of a particle do matter. For a spherical particle of a certain radius (*r*) and dielectric permittivity ($\varepsilon_{p}$) that is immersed in a homogeneous medium of a certain permittivity ($\varepsilon_{m}$) and subjected to a time-averaged electric-field gradient, the averaged dielectrophoretic force for a homogeneous spherical object (like a compact macromolecule) is [28,68]

(32) FDEP=2πr3 Reεp*−εm*εp*+2εm*∇|ERMS→|2

where $\nabla {\left|\vec{E_{RMS}}\right|}^{2}$ is the gradient of the root mean square of the electric field, and Re{…} is the real part of the complex Clausius–Mossotti factor (which determines whether the force is attractive or repulsive towards the regions with the most intense field, i.e., positive or negative DEP, respectively).

In the case of bi-layered objects, with different dielectric permittivity (for the core and the cladding), like a cell or a vesicle, $\varepsilon_{p}$ should be substituted with an effective term defined as

(33) $$\varepsilon_{1eff}^{*}\left(\omega \right)=\varepsilon_{2}^{*}\frac{{\left(\frac{r_{2}}{r_{1}}\right)}^{3}+2\left(\frac{\varepsilon_{1}^{*}-\varepsilon_{2}^{*}}{\varepsilon_{1}^{*}-{2\varepsilon }_{2}^{*}}\right)}{{\left(\frac{r_{2}}{r_{1}}\right)}^{3}-\left(\frac{\varepsilon_{1}^{*}-\varepsilon_{2}^{*}}{\varepsilon_{1}^{*}-{2\varepsilon }_{2}^{*}}\right)}$$

where $\varepsilon_{1}^{*}$ and $\varepsilon_{2}^{*}$ are the dielectric permittivity of the core and the cladding, respectively.

Within biological matter, the standing waves associated with the various kinds of CDs and levels of coherence are the relevant operative agents for chemical and topological ordering. Coherent oscillations persist over time and are confined within the boundaries of the coherence domain giving standing waves spanning spatial ranges of the order of the wavelength λ of the coupled electromagnetic mode, with temporal scales of the order of $1/\omega_{r}$ [62,63,69]. The DEP term force is responsible for the further patterning of matter: the standing waves of confined modes act as a cymatic patterning, creating nodes (where matter accumulates) and antinodes (where matter becomes rarefied), thus, breaking the spatial homogeneity [31,65]. In three dimensions, the superposition of multiple cymatic, dielectrophoretic and ponderomotive contributions generates the typical anisotropic distributions of living matter that take the form of membranes, chains, filaments, vesicles, reticuli, and stacks, precisely the topological motifs observed in living cells and tissues [31,32,43], revealing “order without repetition” (of a lattice unit), as Schrödinger pointed out [70].

The ensemble of QED forces described by Equations (28)–(30), plus the cymatic and dielectrophoretic dynamics (Equations (31)–(33)), provides a physically grounded mechanism by which the coherent water matrix and its associated EM fields drive morphogenesis and impose geometric and stereochemical order upon the molecular constituents of living matter. The frequency-selective nature of the dynamic forces (Equations (29) and (31)) implies that the CD does not interact indiscriminately with all species present in the cellular landscape but rather attracts and orients only those possessing commensurate vibrational modes. Since enantiomeric forms of a chiral molecule possess distinct three-dimensional charge distributions and, consequently, distinct rotational and vibrational spectra (particularly in their coupling to circularly polarized or chiral field configurations), the selective attraction mechanism inherent in the CD field gradients constitutes one of the physical pivots for conceiving the enantiomerically pure synthesis of products.

## 5. Enantiomeric Selection and Supramolecular ‘Optical Molding’: A Possible QED Basis for Chirality

The water-based matrix in living matter is, thus, not a simple solvent but the medium and the agent of a QED field, structured in a hierarchy of oscillation modes which are reasonably endowed with chirality. Indeed, for DNA-hydration shells, it has been shown that the water field has chiral properties and structure [2,70]. The idea proposed here is that the QED background field intertwined with the biological water-based medium (containing also the other molecular species, some of them chiral too) is the agent able to ‘forge’ the molecular structures down to the chirality level (meant in several different senses—from stereoisomers, diastereomers, epimers, and anomers to supramolecular orderings).

Now we have to consider that, in the case of helicoidal structures, the involved EM modes likely have a complex structure of helicity and orbital angular momentum (OAM), beyond the spin angular momentum (SAM) or polarization. In addition, we have to consider how these quantities act on the molecular shaping in the assembly process (for secondary and tertiary supramolecular structures, such as for an alpha-helix or collagen fibers, which have a more complex triply helical shape) and, possibly, even during the synthesis phase for L/R enantiomeric molecular species (see Figure 6 for an intuitive scheme showing the two regimes).

### 5.1. The Case of Helix-like Structures

As shown in Section A Few Examples of Technologies Based on the Chirality of Light, the self-focusing of the EM field in the coherent aqueous matrix generates decohered 1D lanes, whose transverse size is fixed by the effective mass (M) (Equations (16)–(19)), and the boundary condition ($J_{P}=0$) imposes the quantization of the flux threading each lane (Equations (23)–(25)). A phase vortex is, therefore, associated with the axis of every channel: the confined modes acquire an azimuthal phase factor ($e^{il\varphi }$) with integer l, i.e., the very structure that characterizes Laguerre–Gaussian (LG) modes carrying orbital angular momentum (OAM) (lℏ) per photon [1,8,9,11]. Hermite–Gaussian (HG) modes (l= 0), which are superpositions of counter-handed LG pairs [10,15], carry no net OAM and, therefore, cannot exert a net orienting torque on an extended helical object; they can sustain only the spin (SAM) contribution, as discussed in Section 5.2.

When a nascent helical assembly—like, for instance, a polypeptide chain folding into an α-helix or polyproline-II strands winding into the right-handed tropocollagen superhelix [71,72]—develops along such a lane, it behaves as a *helical antenna*. The total time-averaged optical torque (${\vec{\tau }}_{tot}$) [N·m] acting on it decomposes as:

(34) $${\vec{\tau }}_{tot}={\vec{\tau }}_{SAM}^{\left(chiral\right)}+{\vec{\tau }}_{OAM}$$

and, for extended helices, the dominant term is the orbital one:

(35) $${\vec{\tau }}_{OAM}=\Gamma_{l}\;\frac{l\;P_{abs}}{\omega }\;\hat{k}\;=\;\Gamma_{l}\;l\hbar \;\dot{N_{ph}}\;\hat{k}$$

where the helical (geometric) selection rule (which refers to the fact that OAM transfer is only resonant when the azimuthal symmetry of the optical mode ℓ corresponds to that of the molecular structure, *m*, according to the Kronecker delta: $\delta_{l,\;m}$) are according to [27,73,74]

(36) $$\Gamma_{l}={\left|\frac{1}{2\pi }\int_{0}^{2\pi }\chi \left(\varphi \right)\;e^{-il\varphi }\;d\varphi \right|}^{2},\;\;\chi \left(\varphi \right)\propto e^{im\varphi }\;\;\Rightarrow \;\;\Gamma_{l}=\delta_{l,\;m}$$

where ℓ is the topological charge of the confined LG mode (integer, dimensionless; OAM per photon (lℏ) [J·s], with the sign of ℓ defining the handedness of the wavefront, dextrorotatory and levorotatory); $P_{abs}$ is the power absorbed (or scattered) by the forming helix [W = J·s^−1^]; ω is the angular frequency of the mode [rad·s^−1^]; $\dot{N_{ph}}=P_{abs}/\left(\hbar \omega \right)$ is the absorbed-photon rate [s^−1^]; $\hat{k}$ is the propagation unit vector (identical to $\hat{h}$ in the integrated form of the torque used above); $\chi \left(\varphi \right)$ is the normalized azimuthal distribution of the chiral susceptibility of the helix (dimensionless); m is the signed integer number of chiral subunits per turn (m>0 right-handed, m<0 left-handed); and $\Gamma_{l}\in \left[0,1\right]$ is the azimuthal overlap factor (dimensionless).

It is important to highlight that the sign of ℓ defines the ‘hand’ of the helicity of the EM beam, while its numerical value (|ℓ|) is the number of 2π phase wraps that accumulate whilst making a complete revolution around the fixed *z*-axis (total azimuthal phase = 2πℓ), that is, the number of intertwined helices (‘ramps’) of which the wavefront is composed, which is, in turn, the OAM per photon in units of ℏ. Conversely, m is an intrinsic structural property of the matter: a signed integer denoting the number of chiral subunits per turn of the nascent helix, with its sign dictating the macroscopic handedness (right- or left-handed) of the superstructure.

The ansatz $\chi \left(\varphi \right)\propto e^{\left(im\varphi \right)}$ is here stated as a physically motivated phenomenological assumption rather than as a result derived from a microscopic Hamiltonian; its justification rests on three converging arguments. (i) Symmetry: For a helical assembly of pitch P, a translation z → z + P is accompanied by a rigid rotation φ → φ + 2π; the only azimuthal functions consistent with this screw symmetry are the harmonics $e^{\left(im\varphi \right)}$, with m being the number of chiral subunits per turn. The ansatz is, therefore, the symmetry-adapted (lowest-order) form of any azimuthal susceptibility, and m is not a free fitting parameter but a structural integer fixed by the geometry of the helix. (ii) Microscopic support: in a multipolar (dipolar) expansion of the light–matter coupling, the transition matrix element between the confined mode and the nascent helix factorizes into a radial part and an azimuthal overlap integral of exactly the form of Equation (36); for a chain of N subunits at azimuths, $\varphi_{j}=2\pi j/m+l-phase$, the coherent sum $\Sigma_{j}e^{\left(i\left(l-m\right)\varphi_{j}\right)}$ yields $N\cdot \delta_{l,m}$ in the large-N limit, i.e., the Kronecker selection rule emerges from the microscopic arrangement itself. (iii) Consistency: the same integer m appears independently in the structural description of the helix (subunits per turn), so Equation (36) couples a mode property (ℓ) to a matter property (m) without introducing ad hoc parameters. A full derivation of χ(φ) from first principles—including radial and dispersive corrections beyond the dipolar term—is deferred to future work; here, $\chi \left(\varphi \right)\propto e^{\left(im\varphi \right)}$ should be read as the leading symmetry-dictated term of that expansion.

Because of Equation (36), OAM transfer is resonant only when the azimuthal symmetry of the mode matches that of the structure: if the lane sustains predominantly modes with $l=+\left|m\right|$, the corresponding helix receives the torque ($lP_{abs}/\omega$)—the mechanical equivalence demonstrated in the ‘optical spanner’ experiments [75]—which guides and stabilizes its winding. (The mechanical action of OAM was famously demonstrated in a landmark 2001 experiment by Paterson et al. [75], who trapped microscopic absorbing particles in a Laguerre–Gaussian beam. The continuous transfer of OAM from the helical wavefront to the particles exerted a measurable mechanical torque that set them into physical rotation. The authors termed this effect an ‘optical spanner’ because the structured light acted literally like a mechanical wrench, proving that the topological twist of the electromagnetic field can macroscopically ‘twist’ and orient matter). The mirror-image helix ((m→−m) has $\Gamma_{l}=0$) and receives no torque, thus it is not stabilized.

This provides the dynamical mechanism for the uniform handedness of extended supramolecular helices (such as the tropocollagen superhelix or protein α-helices). Physically, this transition is dictated by the size of the assembling structure relative to the wavelength of the confined field: while small, point-like molecules (a≪λ) interact only with the local polarization (dipolar SAM coupling), growing macromolecular assemblies (a≳λ) act as macroscopic helical antennas [73,76]. For these extended objects, the global twisting effect of the beam’s helical wavefront (multipolar OAM coupling) physically overpowers the local spin torque, effectively ‘winding’ the supramolecular structure into its correct macroscopic handedness [1,16].

### 5.2. The Case of Homochirality of Enantiomers

For a point-like enantiomer (an amino acid or a sugar monomer, a≪λ) attracted to the CD boundary by the resonant and ponderomotive forces described in Section 4.3.2, the interaction is dipolar, and chiral discrimination enters through the mixed electric–magnetic (Rosenfeld) component ($\alpha^{\left(chiral\right)}$) of the polarizability tensor, which changes sign between enantiomers ($\alpha_{R}^{\left(chiral\right)}\;=\;-\alpha_{L}^{\left(chiral\right)}$) [1,77]. Two complementary terms arise, formalizing the statements made above that the ‘enantiomer + field mode’ pair is non-degenerate and that the helicity gradient acts as a chiral mold [2,71].

Before detailing the two terms (the thermodynamic and dynamic ones), it is worth making explicit why a helicity gradient necessarily develops at the CD boundary and how it relates to the field gradients described in Section 4.3.2. The self-trapped mode decays exponentially outside the domain ($A(x)=A(0)e^{-cMx/\hbar }$ (Equation (16)), so the confined intensity ($\langle |\vec{E}{|}^{2}\rangle \propto \omega_{CD}^{2}A^{2}$)—and, hence, $\nabla A^{2}$, the driver of the ponderomotive and resonant forces (Equations (27)–(30))—is strongly graded at the interface.

Since the aqueous connectome is already ‘chirally biased’—since the water matrix, templated as a continuum of chiral hydration shells around biomolecules, carries self-trapped coherent EM modes whose helicity sign is necessarily σ≠0 [2,71]—the helicity density, expressible like $h=\sigma (\epsilon_{0}/2\omega_{CD})\langle |\vec{E}{|}^{2}\rangle$ (see why in Appendix A, Appendix A.1), inherits an analogous spatial profile, and a helicity gradient (∇h) coexists with the amplitude gradient of the field ($\nabla A^{2}$):

(37) $$\nabla h=\sigma \;\frac{\epsilon_{0}}{2\omega_{CD}}\;\nabla \langle |\vec{E}{|}^{2}\rangle \;\;\propto \;\;\sigma \;\nabla A^{2}$$

Taking the gradient of the chiral energy shift (Equation (39)) and using the helicity gradient (Equation (37)) yields the enantioselective translational force on the i-th species, which is the formal realization of the ‘chiral Stern–Gerlach deflector’ described in Section A Few examples of Technologies Based on the Chirality of Light (with $\Delta E_{i}$ being the energy difference between the two enantiomers of the i-th species):

(38) $${\vec{F}}_{i}^{\left(chiral\right)}=-\nabla (\Delta E_{i})=\frac{1}{2}\;\alpha_{i}^{\left(chiral\right)}\;\nabla \langle |\vec{E}{|}^{2}\rangle =\frac{\omega_{CD}}{\epsilon_{0}}\;\sigma \;\alpha_{i}^{\left(chiral\right)}\;\nabla h$$

which shares the same spatial driver $\nabla A^{2}$ as $F_{pm}$, $F_{\mathrm{i-}CD}$ and $F_{\mathrm{int}\;\text{1-}2}$ (defined in Section 4.3.2) but, unlike them, changes the sign between enantiomers because $\alpha^{\left(chiral\right)}$ does.

Thus, the gradients at the CD boundary translate into both translational and non-chiral polarizing electrodynamic forces (Section 4.3.2) and chiral actions: a translational enantioselective force (Equation (38)) that enriches the interface with the preferred enantiomers and the rotational torques (Equations (34)–(36), (40) and (41)) that orient and wind them. The local value h (not its gradient) enters, through $\alpha_{ij}^{\left(chiral\right)}$, Equations (39) and (40) below; while the gradient, ∇h, enters Equation (38).

#### 5.2.1. Thermodynamic Terms: Lifting of the Degeneracy

The coupling of $\alpha^{\left(chiral\right)}$ to the helicity-carrying mode confined in the CD removes the energy degeneracy of the two enantiomers:

(39) $$\Delta E=E_{S}-E_{R}=-\frac{1}{2}\;\left\langle {\left|\vec{E}\right|}^{2}\right\rangle \left(\alpha_{ij}^{\left(chiral\right),\;S}-\alpha_{ij}^{\left(chiral\right),\;R}\right)$$

where $\langle {\left|\vec{E}\right|}^{2}\rangle$ is the average intensity of the field confined within the CD [V^2^·m^−2^] and $\alpha_{ij}^{\left(chiral\right)}$ the chiral component of the polarizability tensor ([C·m^2^·V^−1^ ≡ C^2^·m^2^·J^−1^]). Equation (39) is the quantitative form of the lowered activation barrier invoked above; the preferred enantiomer sits on the lower-energy branch of the coupled system, so that the local ratio of the synthesis rates of the two forms scales as $\exp\left(-\Delta E/k_{B}T\right)$ at the interface, a small but decisive bias in the coherent, low-dissipation environment, reinforced at each step of the ordered reaction cycle (see Figure 4). Such a thermodynamic term is very meaningful because it provides a possible consistent physical reason why a specific enantiomer is synthetized instead of its mirror counterpart, being synthesis that occurs in a chirally biased EM background (i.e., one of the coherent water matrices within biological matter). Thus, if the SAM component of torque—as shown below—could appear just to provide the reasons for which different dynamics are involved in two already-existing enantiomers, the thermodynamic term, actually gives us reasons why the synthesis (in a coherent chiral EM field) is ‘upstream’ oriented and selective.

#### 5.2.2. Dynamic Terms: Enantioselective Torque

The same $\alpha^{\left(chiral\right)}$, coupled to the spin angular momentum density of the confined mode, yields a net torque that orients the prochiral groups during the synthesis step:

(40) $${\vec{\tau }}_{SAM}^{\left(chiral\right)}=\frac{2\omega }{\epsilon_{0}}\;\alpha^{\left(chiral\right)}\;{\vec{s}}_{EM}\;=\;\sigma \;\alpha^{\left(chiral\right)}\;\left\langle {\left|\vec{E}\right|}^{2}\right\rangle \;\hat{k}$$

where

(41) s→EM=ϵ02ω ImE*×E+12μ0ω ImB*×B

which is the SAM density of the field [J·s·m^−3^]; σ=H/ℏ=±1 is the sign of the helicity of the CD mode, with $H=S\cdot \hat{k}$ as defined in Section 2.2; $\epsilon_{0}$ is the vacuum permittivity [F·m^−1^]; ω [rad·s^−1^]; and ${\vec{\tau }}_{SAM}^{\left(chiral\right)}$ [N·m]. Equation (40) is the local (field-density) form of the integrated expression ${\vec{\tau }}_{net}\approx \frac{P_{s-f}}{\omega }\;\hat{k}\cdot \left({\vec{\alpha }}_{LL}-{\vec{\alpha }}_{RR}\right)$ used above, upon identifying that $\left({\vec{\alpha }}_{LL}-{\vec{\alpha }}_{RR}\right)\equiv 2\;\alpha^{\left(chiral\right)}$ and $P_{s-f}=c\;u_{EM}\;A_{lane}$ are the powers carried by the confined decohering (and self-focusing) mode, with $u_{EM}=\frac{\epsilon_{0}}{2}\langle |\vec{E}{|}^{2}\rangle$ being the time-averaged EM energy density [J·m^−3^], c the speed of light in vacuum, and $A_{lane}$ the effective transverse area [m^2^] of the decohered lane by the self-focusing (enough energetic) EM field: $A_{lane}\approx \pi (\emptyset /2{)}^{2}$ with ∅≈150 Å, as given by Equation (27). This identity converts the mode-integrated form of the torque, $\vec{\tau }\approx \frac{P_{s-f}}{\omega }\hat{k}\cdot ({\vec{\alpha }}_{LL}-{\vec{\alpha }}_{RR})\;$(where $P_{s-f}/\omega ={\dot{N}}_{ph}\hbar$ is the angular-momentum flux), into the local field-density form of Equation (40). It is the rotational analogue of Equation (39), in the same sense in which chiral forces and chiral energy shifts are two faces of the same light–matter coupling [2,71,78,79]. Because $\alpha^{\left(chiral\right)}$ flips the sign between enantiomers while σ is fixed by the CD mode, the torque acts in the opposite sense on the two specular forms, aligning and activating only the ‘correct’ one.

Both terms (Equations (39) and (40)) scale with the confined intensity $\langle {\left|\vec{E}\right|}^{2}\rangle$ and with the helicity sign (σ) of the mode; they are, therefore, inherited and enormously amplified by the supercoherent hierarchy, as discussed in the following paragraph. In Figure 6, we provide an intuitive scheme for the two main chiral actions of light on biomolecules (helix-like shaping and enantio-selection).

## 6. Conclusions and Perspectives

**Summary**. In light of what has been developed, we now possess the conceptual elements to frame, at least semi-quantitatively, a consistent physical mechanisms underlying the biological shaping of molecules, both in their assembly into secondary and tertiary structures (left-/right-handed α-helices, the triply helical collagen, etc.) and at the deepest level of morphogenesis, homochirality.

We stress that a fully quantitative treatment would require a case-by-case analysis—for each chiral molecular species and for each frequency/wavelength range of the involved EM modes—compounded, for coupled and confined fields, by the fact that the free-photon relation ν = c/λ no longer holds. The present work, therefore, deliberately proposes a semi-quantitative frame: a consistent set of physical processes and scaling relations by which the selecting capacity of the biological medium with respect to chirality (enantiomeric and supramolecular) can be, at least, semi-quantitatively rationalized and, at least in principle, tested.

According to the QED framework of living matter based on its water-matrix-based coherence, the synthesis of any biomolecule—including the chiral ones, such as amino acids, enzymes and sugars—occurs mainly at the boundaries of the many kinds of coherence domains (CDs), where the tails of the self-trapped EM potential generate the most intense gradients and dispersive forces. The reactant molecules are displaced and driven by dielectrophoretic, ponderomotive and frequency-dependent forces that depend on their polarizability and on the resonance between their oscillation modes and those of the CDs present in living matter (made of water and not only).

In the proposed theoretical frame, the coherent EM field trapped in the CD not only would provide energy and encounter timing but would also have non-trivial states of polarization/spin (SAM), chirality/helicity and, in some cases, orbital angular momentum (OAM) to endow it with the capacity to provide a *chiral bias* to the assembly process. This can happen through (i) the non-degeneracy of the energy states of the ‘enantiomer + mode of the EM field’ pair (Equation (39)) and (ii) the enantioselective optical torques (Equations (34)–(36), (40) and (41)) that orient the reactants during the synthesis steps. The local helicity gradient developed by the field at the interface acts, thus, as a sort of ‘chiral mold’ [12,13]: the enantiomer, whose chirality is complementary to the local sign of the helicity, finds a more energetically favorable reaction path, i.e., a lowered activation barrier with respect to its mirror.

The analysis conducted here shows that this chiral molding operates at two distinct levels, governed by the two momenta of light discussed in Section 2 and related, in Appendix B, to the AHK mechanism through which 1D optical-cavity lanes, coupled to the penetration of the fields through the coherent water-based medium, are the loci where such a “molding” occurs.

It has been pointed out the following:

- For *point-like enantiomers* (a≪λ), the interaction is *local* and is carried by the helicity/spin angular momentum (SAM) of the confined mode: the same Rosenfeld pseudoscalar ($R_{0k}$) that determines the optical rotation and the Berry phase (Section 3) builds the mixed electric–magnetic polarizability ($\alpha^{\left(chiral\right)}$), producing both the thermodynamic lifting of the enantiomeric degeneracy (Equation (39)) and the enantioselective SAM torque (${\vec{\tau }}_{SAM}^{\left(chiral\right)}$) (Equation (40));
- For *extended helical assemblies* (a≳λ), the interaction becomes *global*, as in the sense of Figure 1: the decohered 1D lanes generated by AHK self-focusing are themselves optical vortices carrying a quantized flux (Equations (23)–(25)), and, therefore, act as natural cavities for Laguerre–Gaussian modes with topological charge, l≠0.

The OAM torque (Equation (35)), filtered by the helical selection rule (Equation (36)), twists and stabilizes only the superstructure whose azimuthal symmetry matches that of the confined mode, while the mirror-image helix receives no torque. The conservation of the total angular momentum per photon, $j_{z}=(l+\sigma )\hbar$ (Appendix B), thus appears as a possible common root of the two torques and, ultimately, of the two faces of chiral morphogenesis. It is worth stressing that the enantioselective bias (ΔE), although small with respect to $k_{B}T$, acts within a coherent, low-entropy environment [3,30,31,80] and is renewed at each step of the ordered reaction cycle (Figure 4): a kinetic-proofreading-like amplification that renders the bias relevant. This mechanism is enormously amplified by supercoherence [3,7,31,61], that is, where a variety of (hierarchized) nested coherences (on many different degrees of freedom) is in force.

The initial selection of an enantiomer in a CD is transmitted and reinforced throughout the cell through the synchronization of oscillations; once a chiral ‘bias’ is established, the system would tend to maintain and perpetuate it, since many of the newly synthesized molecules will themselves be chiral and will help maintain the coherent field structure that generated them: an autopoietic loop in which structure and function coincide [31,40]. In this picture, the homochirality of life is not the result of a cosmic accident/luck or of a post-synthesis selection (which would be prohibitively expensive) but a direct and necessary consequence of the QED coherence and organization of biological matter: the coherent EM field of the aqueous matrix, with its helicity and angular-momentum properties, acts as a real assembly (analogue) code, guiding molecular synthesis towards specific chiral configurations. Light, therefore, is not only energy for life but an integral part of its syntax [7,31,44,61].

**Perspectives.** The framework proposed here could be falsifiable and some experimental and theoretical pathways could be suggested.

1. Chiral spectroscopy of biological water: chiral sum-frequency generation has already revealed the chiral spine of hydration of DNA [2,71,79]; extending such probes to proteins, membranes and cytoskeleton-like interfaces would test whether the water surrounding homochiral biomolecules carries the helicity sign predicted by Equation (37) (and cf. Equation (A3)).
2. Structured-light experiments: Despite that an in vitro environment does not enjoy the full (super) coherence guaranteed in biological alive systems, a possible test could be made by trying to synthesize model biomolecules (like amino acids, collagen-like peptides, and α-helices) under irradiation—of suitable intensity and spectral range (in IR-UV)—with Hermite–Gaussian versus Laguerre–Gaussian beams with controlled σ and l. This could discriminate the two mechanisms: the SAM/helicity content should bias the enantiomeric excess of point-type chiral species, whereas the sign of l should select the handedness of the supramolecular helix, analogous with the observed ‘optical-spanner’ mechanics [13,75].
3. Isotope and thermodynamic perturbations: still in vitro, comparing the previous experiment with new identical ones where the substitution of H_2_O with D_2_O could—at equal condition—reduce the water-matrix coherence threshold—thus shifting the CD frequencies, $\omega_{CD}$ and the coherent fraction [31]: a correlated degradation of enantiomeric purity and of supramolecular handedness could be expectable.
4. Connection with chiral-induced spin selectivity (CISS): the same chiral coherent interface that selects enantiomers should transfer quasi-free electrons in a spin-selective way [81]; testing spin polarization across CD-like water films (such as against hydrophilic biological surfaces [82]) would link homochirality to the redox efficiency and bioenergetics of coherent water, as discussed in previous works [30].
5. Prebiotic and technological implications: if homochirality emerges from aqueous QED coherence, then prebiotic compartments capable of sustaining coherent water (mineral pores, vesicles, and hydrophilic surfaces) provide a natural scenario for its emergence, and ‘coherent-water-assisted’ synthesis opens a route to enantiopure pharmaceuticals and biomaterials without external chiral catalysts.

Admittedly, the present treatment remains semi-quantitative: a full QFT simulation of the coupled matter–field system at the CD boundary, including the spectrum of confined HG/LG modes and the dynamics of the NG/DWQ condensate, is the necessary next step. Yet the convergence of independent phenomenological threads—the matching of the self-focused lane diameter with the microtubule lumen (Equation (27)), the chiral hydration shells of DNA, the mechanical equivalence of spin and orbital angular momentum, and the geometric-phase nature of optical activity—already provides a consistent, physically grounded possible answer to the question posed in the title: “why homochirality in biology?”.

## Figures and Tables

**Figure 1:**
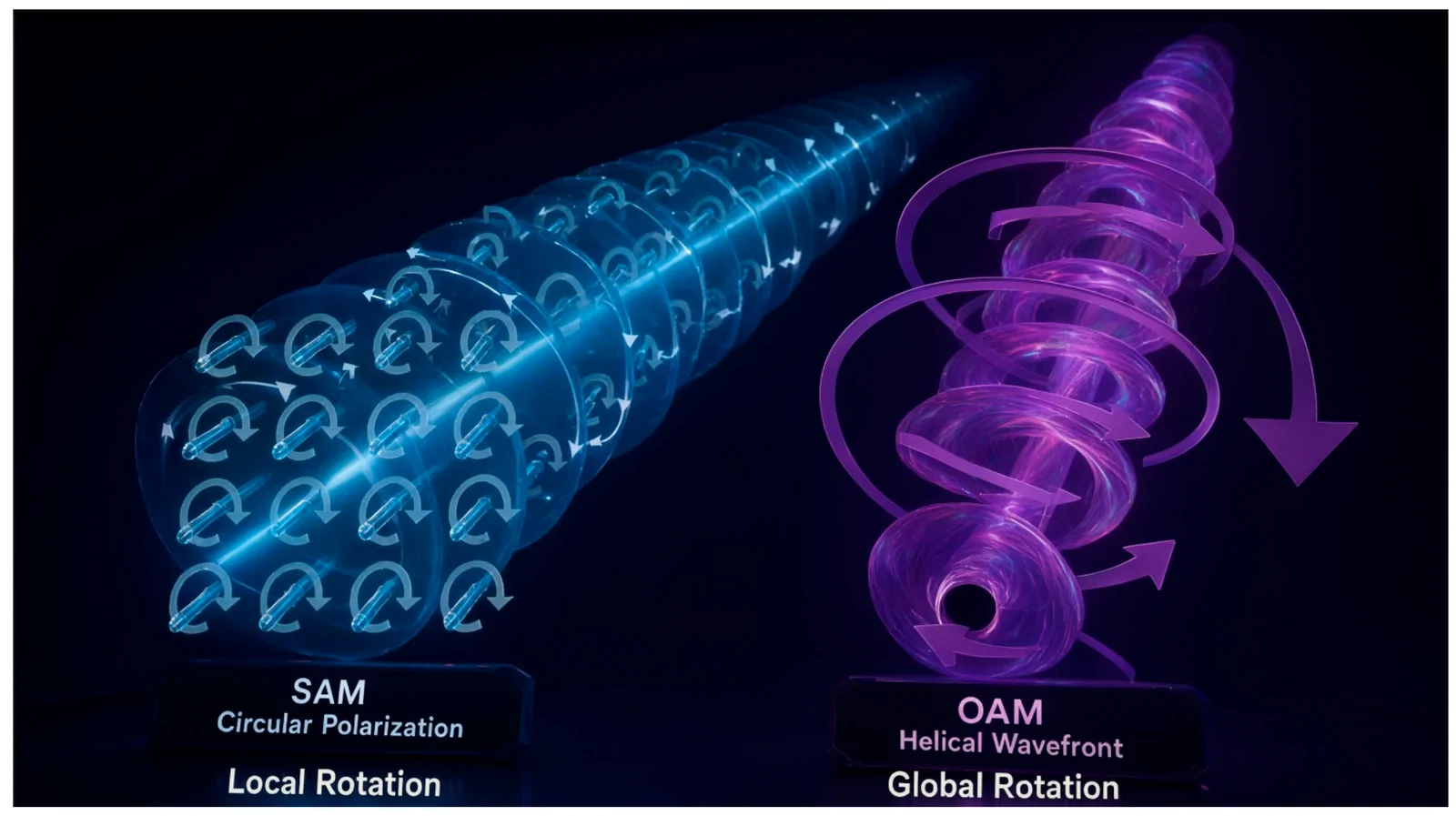
Scheme showing how the rotational transformation of a beam can be performed with two different mappings: on the left, a local rotation, in which the electric field at each position is rotated by a certain angle; on the right, a global rotation, in which the entire complex beam width structure is rotated around a given axis [9].

**Figure 2:**
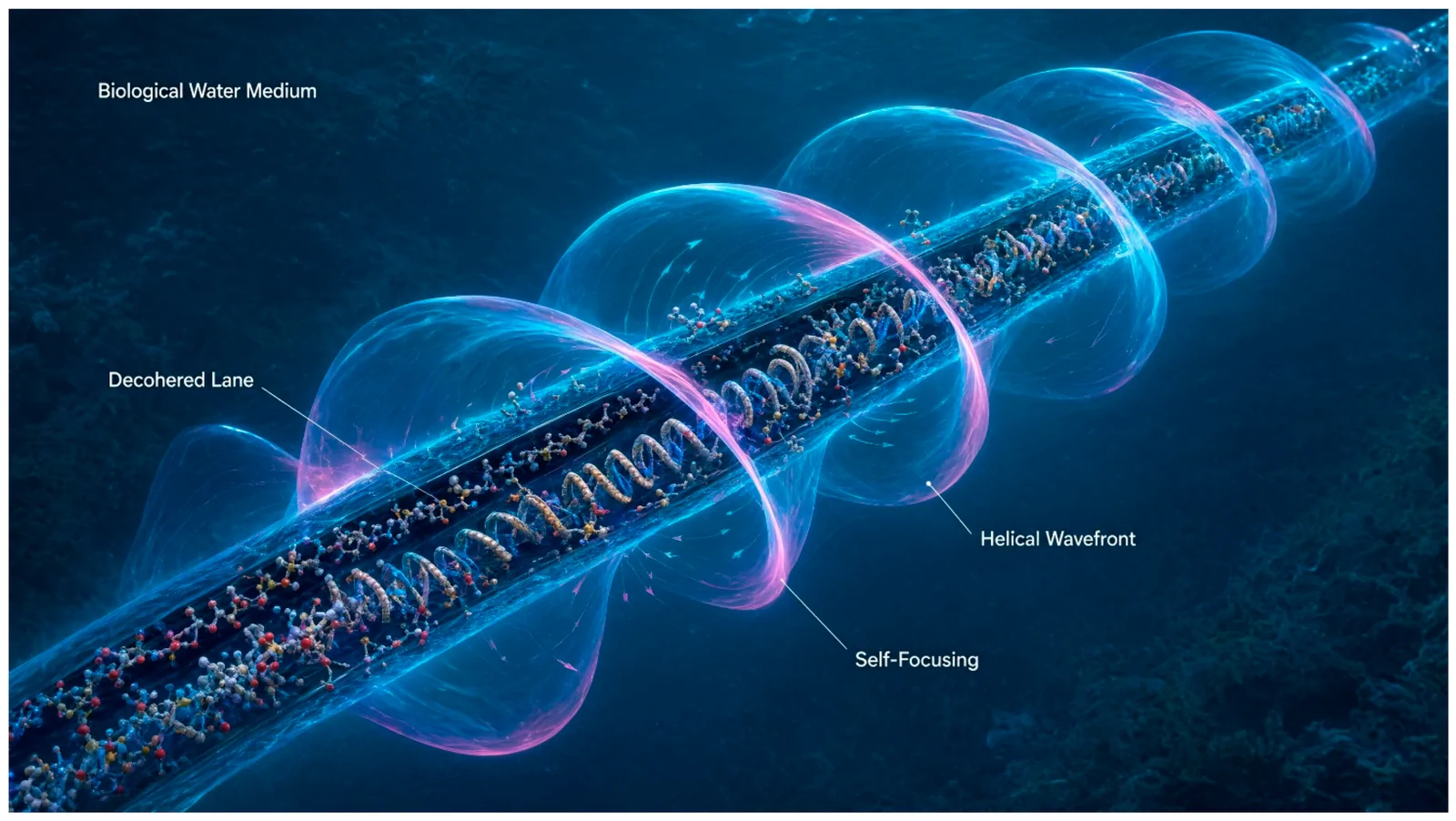
Artistic, non-scaled rendering of a decohered 1D lane carved by the AHK self-focusing of a helical-wavefront (OAM-carrying) electromagnetic mode in the coherent biological water medium: field gradients (for the field, ∇A^2^ and for its helicity density, ∇h) at the lane boundaries attract and chirally mold reactants, while the confined vortex mode twists nascent helical chains into their selected handedness (see Section 4.3, Section 5 and Appendix B for details).

**Figure 3:**
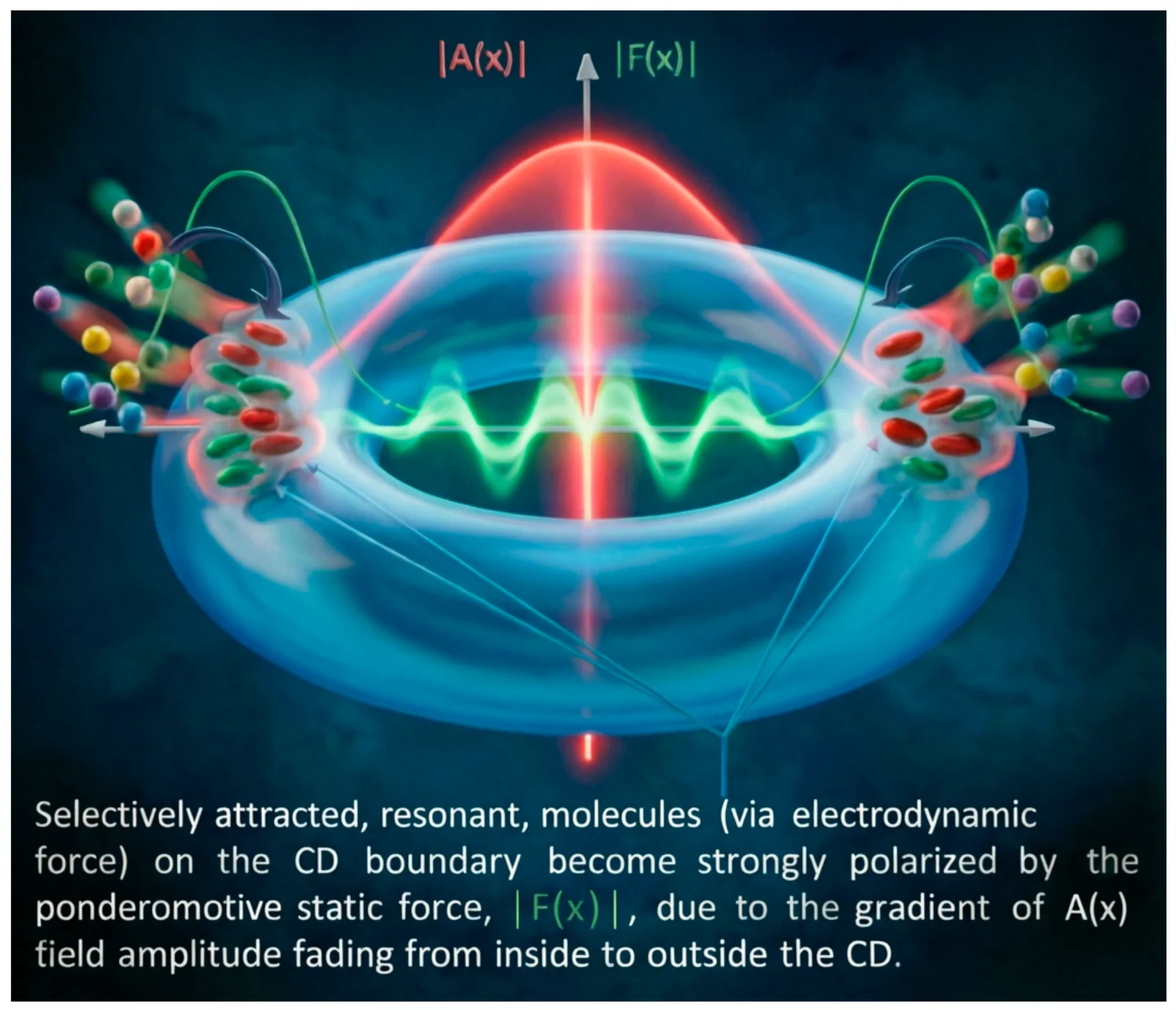
Non-scaled scheme of the two dynamic components (|F(x)|, in green) at the CD interface, generated by the gradient of the electromagnetic potential (|A(x)|, in red). Through the frequency-dependent component, only molecules with oscillation modes resonant with the CD field (red and green spheres) are selectively attracted to the interface. Simultaneously, the frequency-independent component generates a ponderomotive force that repels charges outward. This strongly stretches the electron clouds of the attracted molecules, polarizing them and increasing their reactivity. Such a mechanism facilitates targeted and efficient chemical reactions under the catalytic influence of the CD, explaining how living matter performs highly ordered, rapid biochemical processes with minimal energy expenditure.

**Figure 4:**
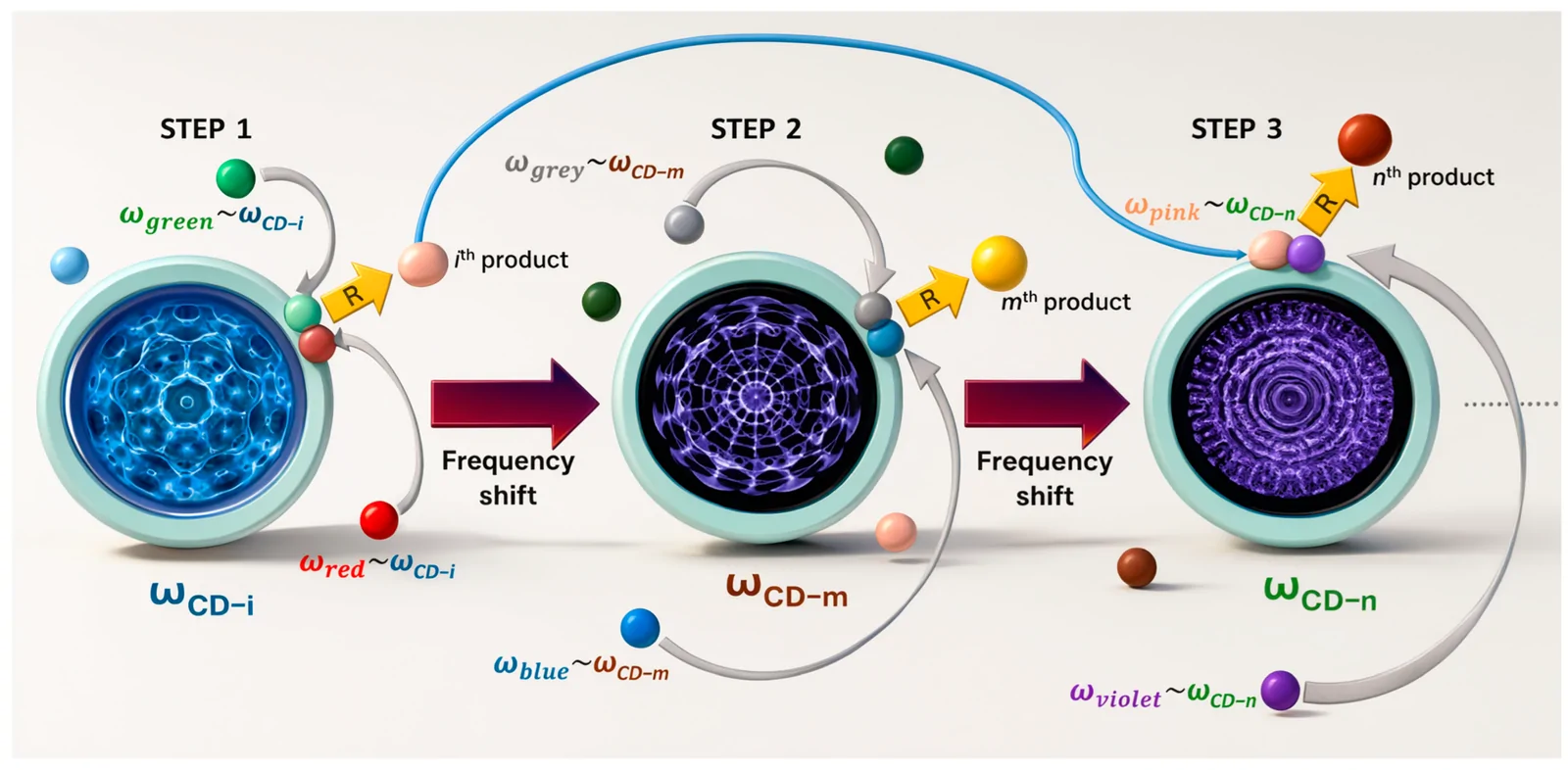
Non-scaled representation showing how QED coherence enables ordered reaction pathways: the operating frequencies (given by the interplay with water and the other biomolecules and ions) determine which chemical encounters occur (and, hence, the form of the assembled matter), while the chemical species present and reaction undertaken, in turn, affect the operating frequencies. At Step 1, the CD oscillating at $\omega_{CD-i}$ selectively recruits resonant species (red and green) and activates them, for example, via quasi-free electron transfer or photon emission matching their electronic/vibrational transitions. This yields two outcomes: (i) a mediated reaction (R) producing the i-th product and (ii) a frequency shift of the CD from $\omega_{CD-i}$ to $\omega_{CD-m}$. The process repeats over many steps until the system could even return to its initial conditions, generating a self-restarting biochemical cycle (as in the Krebs or Calvin cycles). The reaction sequence is, therefore, not random but causally determined by the preceding transitions and teleologically driven by the minimization of the system’s vacuum level [63].

**Figure 5:**
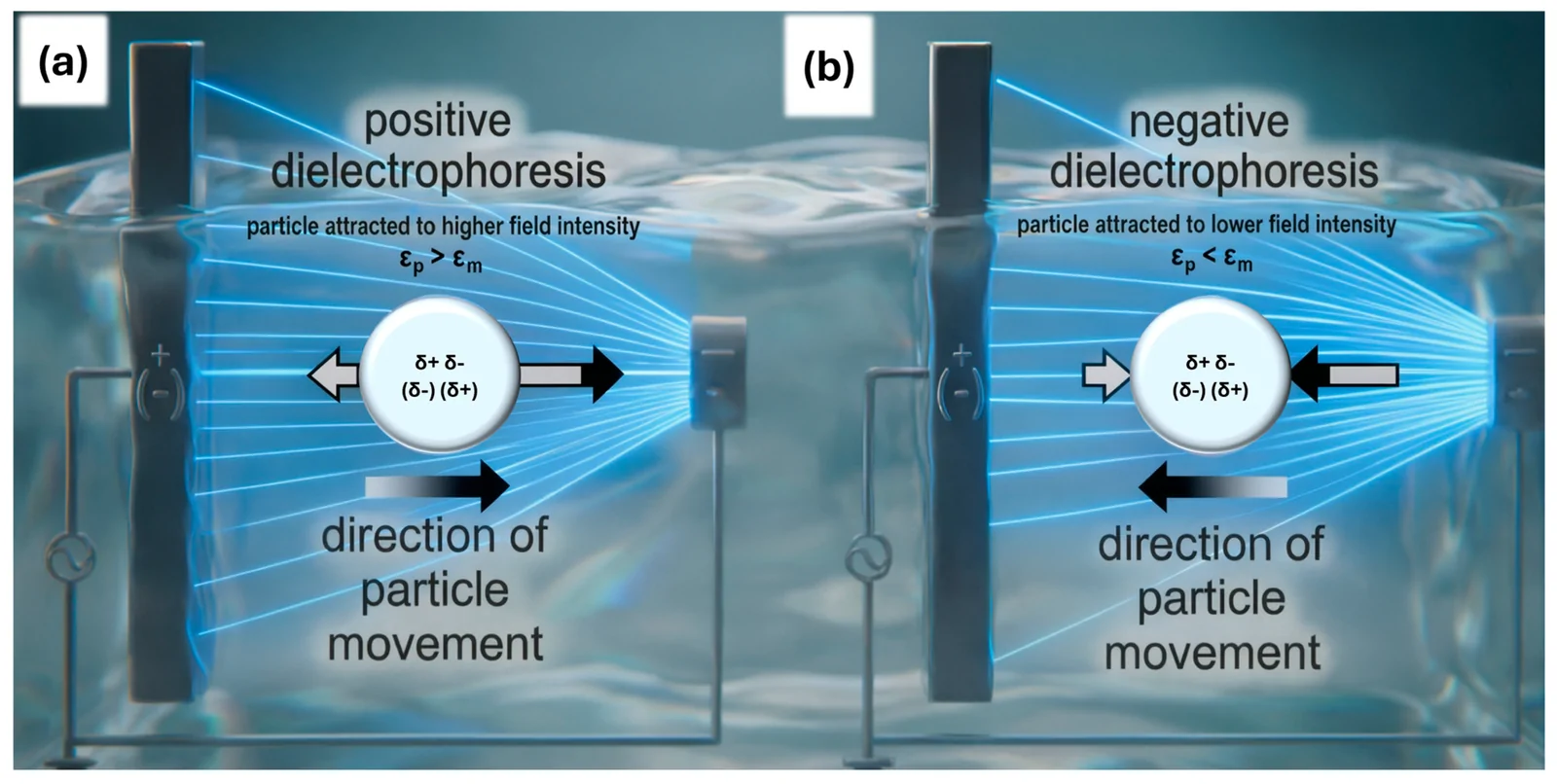
Simplified scheme depicting the two kinds of dielectrophoretic mechanism, where the two force components (for the attraction/repulsion with respect to the inhomogeneous electric field) are unbalanced (represented by non-shaded arrows in (**a**,**b**) panels): on the left (**a**), positive dielectrophoresis (p-DEP) is shown, where the particle, having a higher electrical permittivity than the medium, $\varepsilon_{p}>\varepsilon_{m}$, is driven towards regions with a higher electric-field amplitude; on the right (**b**), negative dielectrophoresis (n-DEP) is shown, where the particle, having a lower electrical permittivity than the medium, $\varepsilon_{p}<\varepsilon_{m}$, is driven towards regions with a lower electric-field amplitude.

**Figure 6:**
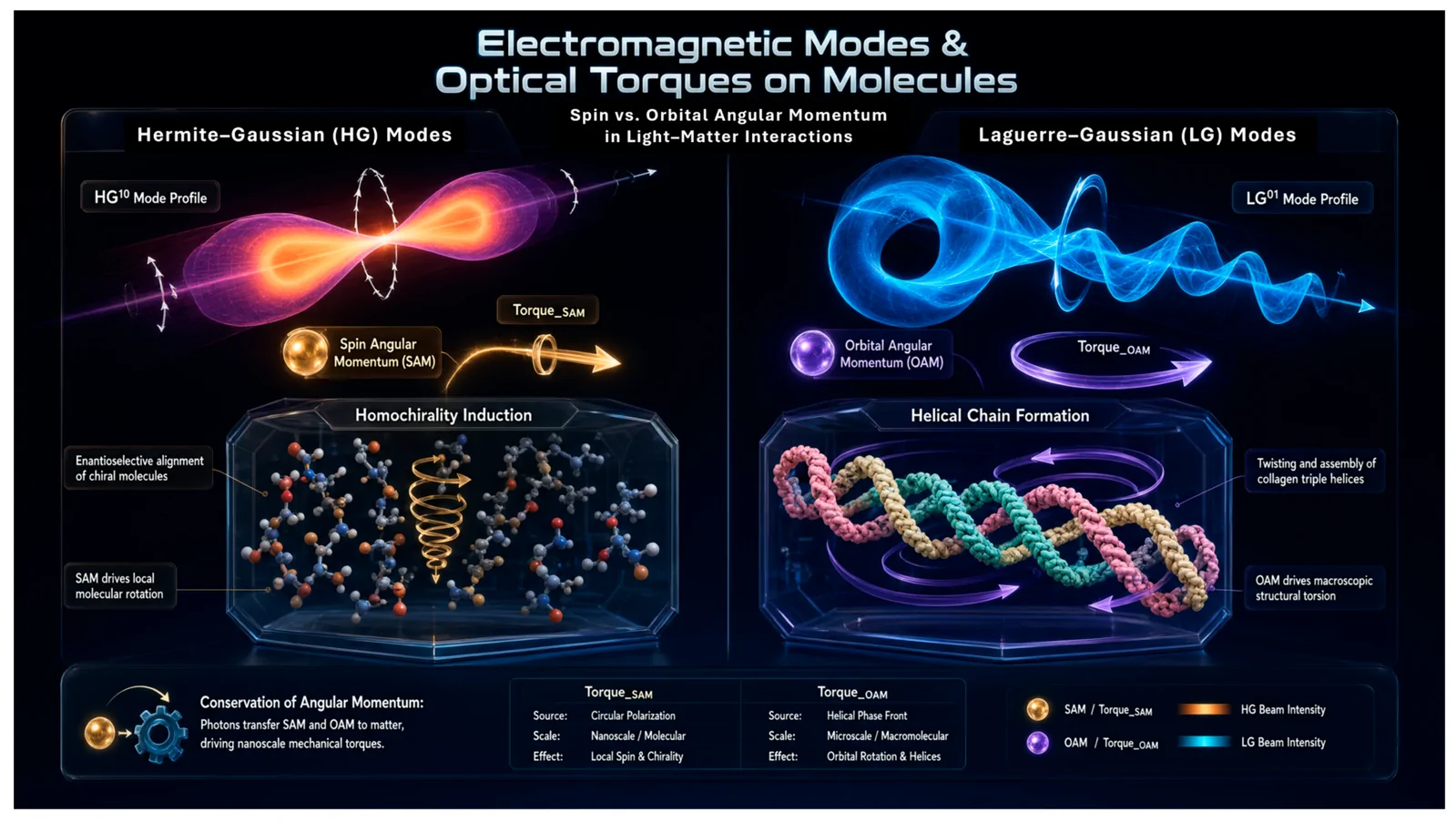
A conceptual scheme showing the two main types of *electromagnetic modes* carrying angular momentum and the optical torques, which they exert on matter within coherent biological microenvironments. Left panel: Hermite–Gaussian (HG) modes, represented by the two-lobed HG_10_ transverse intensity profile, carry spin angular momentum (SAM) associated with the circular polarization of the trapped field. The transfer of SAM to matter generates the spin torque (${\vec{\tau }}_{SAM}^{\left(chiral\right)}$), which operates at the nanoscale/molecular scale, driving local molecular rotation and the enantioselective alignment of chiral species, thereby providing a mechanism for the induction of homochirality within the coherence domains. Right panel: Laguerre–Gaussian (LG) modes, represented by the donut-shaped LG_01_ profile with a helical phase front, carry orbital angular momentum (OAM). The corresponding orbital torque, ${\vec{\tau }}_{OAM}$, acts at the microscale/macromolecular scale, twisting and assembling molecular chains into helical superstructures—as exemplified, for instance, by the collagen triple helices and α-helices—and producing macroscopic structural torsion. In the bottom panel a conceptual synthesis is provided for the conservation of angular momentum in light–matter interactions: photons transfer the conserved total angular momentum ($J_{z}$) to matter, driving nanoscale mechanical torques SAM and OAM. The table summarizes, for each torque, the source (circular polarization vs. helical phase front), the scale of action (nanoscale/molecular vs. microscale/macromolecular), and the resulting structural effect (local spin and chirality vs. orbital rotation and helix formation). The color code in the legend identifies SAM/${\vec{\tau }}_{SAM}^{\left(chiral\right)}\;$(gold), OAM/${\vec{\tau }}_{OAM}$ (violet), and the intensity distributions of the HG (orange) and LG (blue) beams. Note that within the tightly confined 1D lanes, spin–orbit coupling mixes the local spin and orbital degrees of freedom. Therefore, the separation into distinct SAM and OAM channels depicted here is to be understood as an effective phenomenological mapping that clarifies the distinct biological mechanisms (enantioselection vs. helical molding), while the underlying physical drive remains the exact conservation of the total angular momentum ($J_{z}$) (see Appendix B).

## Data Availability

No new data were created or analyzed in this study. Data sharing is not applicable to this article.

## Abbreviations

The following abbreviations are used in this manuscript:

QED	Quantum Electrodynamics
QFT	Quantum Field Theory
QM	Quantum Mechanics
SSB	Spontaneous Symmetry Breaking
NG	Nambu–Goldstone
CD	Coherence Domain
DW	Dipole Wave Quanta
DEP	Dielectrophoresis
SAM	Spin Angular Momentum
OAM	Orbital Angular Momentum

## Appendix A. Zilch Tensors and Helicity

Zilch tensors (often referred to in the plural as *Zilches*) are a set of conserved quantities in the electromagnetic field in vacuum, introduced by Daniel M. Lipkin in 1964 [83]. Unlike the energy-impulse tensor, which describes the transport of energy and momentum, Zilch tensors represent additional conserved quantities related to the internal structure, chirality, and topological properties of electromagnetic field lines. Some key points about Zilch tensors are summarized in the following:

### Appendix A.1. Definition and Mathematical Properties

The Zilch tensor, often denoted as $Z^{\mu \nu \rho }$ (or sometimes as $Z^{abc}$), is a rank 3 tensor constructed from the electromagnetic field tensor $F^{\mu \nu }$ and its Hodge dual $⋆F^{\mu \nu }$ (defined using the Levi–Civita symbol $\left(\epsilon^{\mu \delta \alpha \beta }\right)$ as $\left(⋆F^{\mu \delta }=\frac{1}{2}\epsilon^{\mu \delta \alpha \beta }F_{\alpha \beta }\right)$). It satisfies a local conservation law ($\partial_{\rho }Z^{\mu \nu \rho }=0$), which implies that its spatial integral (the Zilch charge) is independent of time. It is symmetrical in the first two indices ($Z^{\mu \nu \rho }=Z^{\nu \mu \rho }$) and a zero-track tensor. Last, it can be expressed as

(A1) $$Z^{\mu \nu \rho }=⋆F^{\mu \delta }F_{\delta ,}^{\nu \rho }-F^{\mu \delta }⋆F_{\delta }^{\nu \rho }$$

highlighting the link with the derivatives of the field. Lipkin identified ten independent conserved quantities (or rather, nine independent ones, being part of a four-dimensional symmetric-traceless rank 3 tensor, sometimes reduced to 10 fundamental components).

The temporal component $Z^{000}$ of the tensor—the so-called ‘00-zilch’ (zilch density) [83]—is related to optical chirality, which measures the specular asymmetry (right-handed or left-handed) of light: up to a conventional constant, it coincides with the optical chirality density (C)—defined below in Appendix A.2—of Tang & Cohen [78], with its density being proportional to

(A2) $$E\cdot (\nabla \times E)+c^{2}\;B\cdot (\nabla \times B)=\frac{2}{\epsilon_{0}}C$$

and circularly polarized waves are eigenstates of the Zilch tensor. For strictly monochromatic fields, the cycle-averaged 00-zilch reduces to $\omega^{2}\langle h\rangle$ (as made explicit by Equation (A4) below).

Relating this to what is given in Section 2.2—defining $H=S\cdot \hat{k}$, whose eigenvalues given by σℏ=±ℏ with σ=H/ℏ=±1, for purely circularly polarized waves—it is important to point out that the total helicity is the volume integral of the *helicity density* (h), $H=\int_{V}h\;d^{3}x$, whose time-averaged monochromatic form is

(A3) h=− ϵ0c2ω ImE*⋅B = σ uEMω=  σϵ02ω⟨∣E→∣2⟩ J⋅s⋅m−3

with $u_{EM}=\frac{\epsilon_{0}}{2}\langle |\vec{E}{|}^{2}\rangle$ being the confined energy density used in Equations (39) and (40) (same amplitude convention). Hence, h is a genuine angular-momentum density, and for a monochromatic plane wave, it coincides with the projection onto $\hat{k}$ of the SAM density (${\vec{s}}_{EM}$) appearing in Equation (40), the continuum ‘field theory’-counterpart of $H=S\cdot \hat{k}$. This means that such a continuous field density, which generates the mechanical SAM-torque (in Section 5.2), is the exact field counterpart of the quantum spin of a single photon; like to say that it is not an ad hoc postulated classical force: the continuous macroscopic torque that shapes biological helices (Equations (35) and (36)) is the macroscopic, continuous and spatial manifestation of the most intimate and fundamental quantum property of light: the helicity of the single photon (±ℏ).

To make the coupling ‘bookkeeping’ fully explicit, we summarize which quantity enters which physical effect, and with which dimensions. (i) The **local value** of the helicity density (**h**) [J·s·m^−3^]—equivalently, the optical chirality density $C\;=\;(\omega^{2}/c)h$ [J·m^−4^]—is the quantity that couples to the molecular pseudoscalar α^(chiral) in the enantioselective energy shift (Equation (39)) and in the SAM torque (Equation (40)); this is the standard structure of chiral optical forces [1,77]. (ii) The **gradient** ∇h [J·s·m^−4^] (Equations (37) and (A5)) is the quantity that couples to $\alpha^{\left(chiral\right)}$ in the enantioselective translational force (Equation (41)), i.e., the chiral Stern–Gerlach mechanism. (iii) The **00-zilch** Z^000^ [J·s^−1^·m^−3^] does not enter any of the above couplings: for monochromatic fields, it is proportional to h (Equation (A4)), but for polychromatic or structured fields—such as the vortex modes of the decohered lanes—it decouples from h and C, and being P-odd/T-even only in the monochromatic limit while being T-odd in general (Table A1), it cannot shift a thermodynamic equilibrium between enantiomers. Hence, throughout this work, ‘the quantity that couples to matter’ is h (or, equivalently, C) locally and ∇h translationally; Z^000^ is retained only as a conserved ‘bookkeeping’ quantity of the free field.

With the field expressions of Equations (A2) and (A3) and the optical chirality density C [J·m^−4^], the comparison among the three pseudoscalar densities can be made precise: for strictly monochromatic fields they are related by the dimensionally consistent chain $\left\langle Z^{000}\rangle =c\;\langle C\rangle =\omega^{2}\;\langle h\right\rangle$ and per photon:

(A4) $$H\to \sigma \hbar ,\;\;\;\;C\to \sigma \frac{\hbar \omega^{2}}{c},\;\;\;\;Z^{000}\to \sigma \hbar \omega^{2}$$

Equation (A4) makes explicit why the distinction between the two quantities has historically been obscured: for strictly monochromatic light the density of $Z^{000}$ is directly proportional to the helicity density ($Z^{000}=\omega^{2}h$), and this has led to the mistaken zilch for helicity in the past. However, in general cases or for polychromatic fields, there is no simple relationship between the two quantities, and they should not be considered synonyms. In particular, for polychromatic or structured fields—such as the vortex modes confined in the decohered lanes, discussed in Appendix B—the three quantities of Equation (A4) decouple and must not be used interchangeably; the physically operative one in the enantioselective coupling (Equations (40) and (41)) is h (or, equivalently, C), not $Z^{000}$.

The difference between helicity and the Zilch tensor (especially the variants mentioned as *ab-infra-zilch* and *00-zilch*) is, therefore, subtle but substantial, covering their physical nature, the symmetries from which they derive, and their dimensions.

1. Physical nature and dimensions.
  - Helicity H (or its density h): It is considered a true intrinsic angular momentum; it has the dimensions of an angular momentum [J·s] (its density [J·s·m^−3^]) and its value is equivalent to σℏ per photon. It is a P-odd/T-even pseudoscalar — a faithful measure of the ‘true’ chirality of light in Barron’s sense [12,13] — and the total helicity ($H=\int h\;d^{3}x$) is a Lorentz-invariant pseudoscalar.
  - Zilch ($Z^{000}$ and variants): (i) the *ab-infra-zilch* is a component of a rank 2 pseudotensor and not a simple scalar; (ii) $Z^{000}$, discovered before helicity, is **not** an angular momentum, as it does not have the correct physical dimensions ($[Z^{000}]=$ J·s^−1^·m^−3^), and its value per photon is equivalent to $\sigma \hbar \omega^{2}$ (where ω is the angular frequency), precisely the $\omega^{2}$ factor appearing in Equation (A4).
2. Origin of symmetry (Noether’s theorem): Both quantities are conserved and associated with symmetries of Maxwell’s equations, but the transformations are different.
  - Helicity conservation derives from the symmetry of ‘electric–magnetic democracy; (dual rotation), the same transformation $\left\{E\to E+\theta B,\;\;B\to B-\theta E\right\}$ (in units with c=1) introduced in Section 2.2, which rotates the electric and magnetic field vectors into each other around the direction of the wave vector ($\hat{k}$), with a pseudoscalar angle (θ).
  - The conservation of the ab-infra-zilch is associated with a more complex rotation of the field vectors across a tensor angle ($\theta_{ab}$).
  - The conservation of the 00-zilch is associated with a symmetry involving the second time derivatives of the fields ($\left(\ddot{E}\right)$ and $\left(\ddot{B}\right)$) and does not qualify as a simple geometric rotation, since the transformation parameter does not have the dimensions of an angle.
3. Relativistic behavior: while the total helicity is a Lorentz-invariant pseudoscalar, the 00-zilch is only a component of a tensor and is not invariant under complete rotations in spacetime (said ‘boosts’). Table A1 offers a short summary of the differences among the three quantities.

**Table A1:** Helicity, optical chirality and 00-zilch compared by the per-photon value, dimensions, p/t behavior (true vs. false chirality in Barron’s sense) and Noether symmetry of origin.

Quantity	Symbol	Per Photon	Dimensions	p/t Oddness or Evenness	Noether Symmetry
**Helicity** **(its density)**	H (h)	σℏ	J·s·m^−3^	p-odd/t-even	Dual rotation
**Optical** **chirality**	C	$\sigma \hbar \omega^{2}/c$	J·m^−4^	p-odd/t-even	Dual rotation (for monochromatic)
**00-Zilch**	$Z^{000}$	$\sigma \hbar \omega^{2}$	J·s^−1^·m^−3^	p-odd/t-odd (“false chirality”)	2nd-Derivative symmetry

This distinction is not merely a mathematical curiosity but has profound thermodynamic consequences for biological homochirality. According to Barron’s classification of chiral influences [13,25], only t-even pseudoscalars (‘true chirality’) can induce a thermodynamic enantiomeric excess in equilibrium, because they can lift the energy degeneracy between enantiomers without violating time-reversal symmetry (Curie’s principle and Kramers’ theorem) [84,85]. Conversely, t-odd pseudoscalars (‘false chirality’, such as a static magnetic field or the $Z^{000}$ tensor itself) cannot shift the thermodynamic equilibrium between specular forms, although they may affect kinetic reaction rates. This is precisely why, in the QED framework, the T-even helicity (H) and optical chirality *(*C)—and not the 00-zilch—are identified as the agents capable of generating the thermodynamic energy shift for the two enantiomers (Equation (39)) and the enantioselective torque (Equation (40)) that drive biological homochirality.

Finally, the helicity density of Equation (A3) is the quantity from which the ‘chiral mold’ of Section 5.2 derives. Recalling Equation (37), the spatial gradient of the helicity density, specialized to the trapped CD mode, is obtained by imposing the helicity-eigenstate condition B=−iσE/c of the coupled field: the factor c of Equation (A3) cancels exactly against the 1/c inherent in that eigenstate relation, and since σ and ω are spatially uniform for the confined mode, the entire spatial structure—and, hence, the chiral force—is carried by ∇A^2^ (Equation (16)).

Within CDs, $\nabla h=\sigma \frac{\epsilon_{0}}{2\omega_{CD}}\nabla \langle |\vec{E}{|}^{2}\rangle$, and, since $\langle |\vec{E}{|}^{2}\rangle \propto \omega_{CD}^{2}A^{2}$, the helicity gradient results in

(A5) $$\nabla h=\sigma \;\frac{\epsilon_{0}\;\omega_{CD}}{2}\;\nabla A^{2}\left[J\cdot s\cdot m^{-4}\right]$$

with $\nabla A^{2}$ from Equation (16) (the dimensions of the helicity gradient are respected in both forms).

In summary, helicity is a fundamental measure of chirality related to the rotation between the fields E and B, while zilch (in its various forms) is a conserved quantity related to higher-order derivatives or tensor structures; although related to chirality in certain contexts (such as circular dichroism), it differs from helicity dimensionally and geometrically [1].

### Appendix A.2. Applications and Relevance

Recently, interest in Zilch tensors has been revived [10], like the optical chirality described by $Z^{000}$ which is crucial in the study of light–matter interaction with chiral structures (enantiomers, plasmonic nanostructures). Operationally, the quantity that enters the chiral light–matter coupling is the helicity density h (or, equivalently, the optical chirality density $C=\frac{\omega^{2}}{c}\;h$ [J·m^−4^] given in [77]): the enantioselective energy shift and torque of Equations (39) and (40) scale as $\alpha^{\left(chiral\right)}\;h$, i.e., as the product of the molecular pseudoscalar and the field helicity density, i.e., the standard structure of chiral optical forces [1,77].

Moreover, Zilch tensors can encode information about the topology of the lines of force of the electromagnetic field. They derive from a ‘Noether’ symmetry of Maxwell’s Lagrangian, specific for duality symmetry (rotation between electric and magnetic fields), distinct from Poincare symmetries related to impulse energy, and there are extensions of Zilch tensors to general relativity (linearized gravity). In summary, Zilch tensors provide a deeper description of the ‘handedness’ or chiral properties of the electromagnetic field that are not captured by classical energy-pulse tensors.

## Appendix B. AHK Self-Focus Lanes as Optical-Vortex Cavities: Flux Quantization, OAM Modes and $J_{z}$ Conservation as the Common Origin of the Enantioselective and Helical-Shaping Torques

It is worth stressing that the expression of the topological charge defined as

(A6) $$l=\frac{1}{2\pi }\oint_{C}\nabla_{⊥}\Phi \cdot dl\;\;\in \;\;Z$$

has the same topological origin as the flux quantization of Equations (23)–(25) (of the field self-focusing):

- There, the single-valuedness of the dipole-condensate phase θ enforces $\frac{1}{2\pi }\oint \nabla \theta \cdot ds=n$;
- While here, the single-valuedness of the optical phase (Φ) enforces $\frac{1}{2\pi }\oint \nabla_{⊥}\Phi \cdot dl=l$.

The two integers, n and l, are, therefore, two faces of the same requirement. Consequently, the decohered 1D lane produced by AHK self-focusing—which carries a quantized flux (n) and a phase singularity on its axis—is a natural cavity for modes with l≠0: the channel is itself an optical vortex, and the confined field inherits the azimuthal structure $e^{il\varphi }$. This is exactly the l that reappears in the OAM torque (Equation (35): ${\vec{\tau }}_{OAM}$) and in the helical selection rule (Equation (36)).

Because the 1D lane possesses cylindrical symmetry, by Noether’s theorem, the total angular momentum ($J_{z}$) is an exact constant of motion (remains a “good quantum number”), regardless of whether the system is paraxial or not (The ‘paraxial regime’ formally requires $|k_{⊥}|\ll k_{z}$. For the AHK lanes with transverse size ∅≈150 Å, the strong confinement yields|$k_{⊥}$|~2π/∅ ≈ 4.2 × 10^8^ m^−1^. For the electronic CD mode (λ ≈ 100 nm, $k_{z}$ ≈ 6.3 × 10^7^ m^−1^), the ratio |$k_{⊥}$|/$k_{z}$ ranges about from 32 to 700; thus, for these tightly confined 1D lanes, the strict paraxial approximation is violated, and spin–orbit coupling is significant. However, the total angular momentum ($J_{z}$) is an exact constant of motion for cylindrically symmetric fields, independent of the paraxial limit). The eigenvalue of the total angular momentum per photon is $j_{z}\hbar$.

In the paraxial limit (e.g., for larger, less-confined supramolecular structures), $J_{z}$ cleanly separates into orbital and spin contributions, allowing one to write $J_{z}=L_{z}+S_{z}$ and $j_{z}=(l+\sigma )\hbar$—with σ=H/ℏ=±1, as defined in Section 5.2. However, in the non-paraxial regime of the tightly confined 150 Å lanes, spin–orbit coupling prevents the separate conservation of $L_{z}$ and $S_{z}$, and the strict identification $j_{z}=l+\sigma$ no longer holds for the exact eigenstates of the field. Nevertheless, the total $J_{z}$ remains strictly conserved, and its total eigenvalue $j_{z}\hbar$ is well-defined.

The conservation of $J_{z}$ upon absorption of the mode is the common origin of the macroscopic torque on matter of Section 5, since the torque is, by definition, the rate of angular-momentum transfer from the EM field to matter. The time-averaged torque ($\tau_{z}$) [N·m] around the propagation axis *(*$\hat{k}$), is just:

(A7) $$\tau_{z}=\frac{d\left\langle J_{z}\right\rangle }{dt}={\dot{N}}_{ph}\;j_{z}=j_{z}\frac{P_{ext}}{\omega }$$

where ${\dot{N}}_{ph}=P_{ext}/(\hbar \omega )$ the extinction rate of photons [s^−1^]; $P_{ext}=P_{abs}+P_{scat}$ the extinguished power [W]. Since ℏ cancels out, the torque depends only on classical quantities and the total topological charge ($j_{z}$) [27,59].

To connect this exact macroscopic result to the specific biological mechanisms described in Section 5, we adopt an **effective paraxial interpretation**: we map the total torque into a spin-dependent (enantioselective) part and an orbital-dependent (helical-molding) part by formally substituting $j_{z}\to l+\sigma$. Under this phenomenological mapping, the σ-proportional term yields the enantioselective SAM torque (Equation (40): ${\vec{\tau }}_{SAM}^{\left(chiral\right)}$), while the lℏ-per-photon part yields the ‘helical-molder’ ${\vec{\tau }}_{OAM}$.

In local form, for the enantio-selectivity, with an intensity (EM energy flux per time unit) of $I=\frac{c\epsilon_{0}}{2}\langle |\vec{E}{|}^{2}\rangle$ and extinction cross-sectional area ($A_{ext}$) [m^2^], the exact total torque is defined as

(A8) $$\tau_{z}=(l+\sigma )\frac{c\epsilon_{0}}{2}\langle |\vec{E}{|}^{2}\rangle A_{ext}/\omega$$

and exhibits the same scaling with the confined intensity as Equations (39) and (40). In the effective paraxial interpretation, the two addends would map exactly onto the two distinct mechanisms described in Section 5:

- $lP_{ext}/\omega$ correspond to the helical-molding (${\vec{\tau }}_{OAM}$) (weighted by $\Gamma_{l}$, Equation (36));
- $\sigma P_{ext}/\omega$ corresponds to the enantioselective (${\vec{\tau }}_{SAM}^{\left(chiral\right)}$) (it is the integrated form of Equation (40)).

In the non-paraxial regime of the 1D lanes, spin–orbit coupling mixes the local spin and orbital degrees of freedom. However, the **total torque** (Equation (A7)) remains exactly quantized and drives the chiral/helical selection. The separation into distinct SAM and OAM channels discussed in Section 5 is, therefore, to be understood as an effective phenomenological picture that would be strictly mathematically additive only in the paraxial limit (e.g., for larger propagation lanes) [8,15].
